# Cancer-Related Mutations in the Sam Domains of EphA2 Receptor and Ship2 Lipid Phosphatase: A Computational Study

**DOI:** 10.3390/molecules29051024

**Published:** 2024-02-27

**Authors:** Marian Vincenzi, Flavia Anna Mercurio, Ida Autiero, Marilisa Leone

**Affiliations:** Institute of Biostructures and Bioimaging, Via Pietro Castellino 111, 80131 Naples, Italy; marian.vincenzi@ibb.cnr.it (M.V.); flaviaanna.mercurio@cnr.it (F.A.M.); ida.autiero@cnr.it (I.A.)

**Keywords:** Sam domain, EphA2, Ship2, Mid Loop (ML), End Helix (EH), AlphaFold2, molecular dynamics, cancer

## Abstract

The lipid phosphatase Ship2 interacts with the EphA2 receptor by forming a heterotypic Sam (sterile alpha motif)–Sam complex. Ship2 works as a negative regulator of receptor endocytosis and consequent degradation, and anti-oncogenic effects in cancer cells should be induced by hindering its association with EphA2. Herein, a computational approach is presented to investigate the relationship between Ship2-Sam/EphA2-Sam interaction and cancer onset and further progression. A search was first conducted through the COSMIC (Catalogue of Somatic Mutations in Cancer) database to identify cancer-related missense mutations positioned inside or close to the EphA2–Sam and Ship2–Sam reciprocal binding interfaces. Next, potential differences in the chemical–physical properties of mutant and wild-type Sam domains were evaluated by bioinformatics tools based on analyses of primary sequences. Three-dimensional (3D) structural models of mutated EphA2–Sam and Ship2–Sam domains were built as well and deeply analysed with diverse computational instruments, including molecular dynamics, to classify potentially stabilizing and destabilizing mutations. In the end, the influence of mutations on the EphA2–Sam/Ship2–Sam interaction was studied through docking techniques. This in silico approach contributes to understanding, at the molecular level, the mutation/cancer relationship by predicting if amino acid substitutions could modulate EphA2 receptor endocytosis.

## 1. Introduction

**Figure 1:**
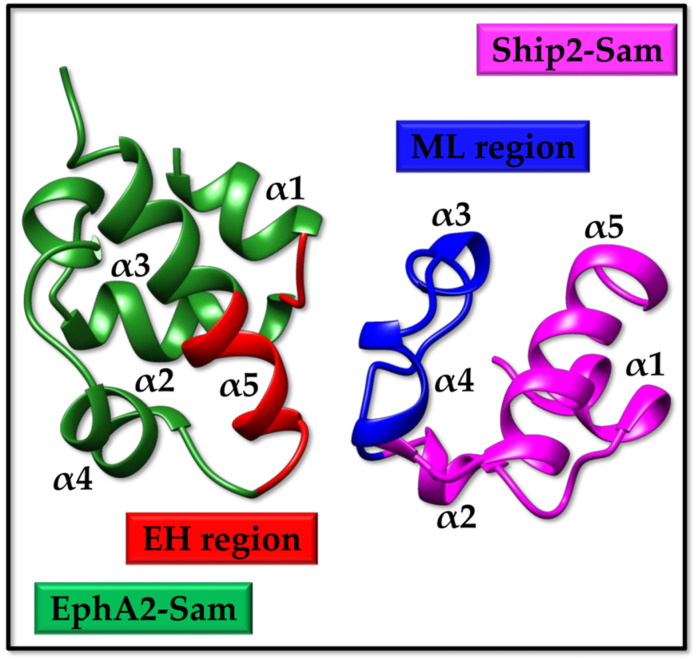
The EH/ML model of the EphA2–Sam/Ship2–Sam complex (pdb entry 2KSO [16], first conformer). The EH and ML interfaces in EphA2–Sam (residues I916-M918 and P952-Y960) and Ship2–Sam (residues H1219-E1238) are coloured in red and blue, respectively.

EphA2 is a receptor tyrosine kinase that is linked to several physiological and pathological processes [1,2]. Recent studies have pointed out that EphA2 is involved in the regulation of lens transparency, kidney reparation following renal damage, the development of the inner ear, and bone remodelling [1]. On the other side, EphA2 is related to several diseases, including osteoporosis [3], cataracts [4], viral infections [5,6,7], and, above all, cancer [8]. The role of EphA2 in cancer appears complex and controversial as receptor-induced effects in cancer cells derive from the fine tuning of an anti-oncogenic ephrin ligand-dependent pathway and a pro-oncogenic ligand-independent lane [9,10]. EphA2 levels are high in many types of tumours, including but not limited to prostate, breast, kidney, glioblastoma, and melanoma, and this upregulation correlates with a very aggressive cancer phenotype and poor survival [11,12]. Therefore, ligand-induced EphA2 receptor endocytosis and subsequent degradation attract attention in the drug discovery field as a possible path to lower tumour malignancy [13]. In this context, the lipid phosphatase Ship2 also assumes a certain relevance in working as a negative modulator of receptor endocytosis [13]. Ship2 is engaged by EphA2 through heterotypic Sam (sterile alpha motif)–Sam interaction [13]. Sam domains are small protein binding modules with a helical fold that are very versatile, concerning both functions and binding properties. Sam domains exhibit a tendency to self-associate, forming homo- and hetero-dimers, oligomers, and even polymers [14]. The interaction between the Sam domains of both EphA2 (EphA2-Sam) and Ship2 (Ship2-Sam) has been precisely characterized (Figure 1) [15]. Structural studies and interaction assays with an array of biophysical techniques have revealed that the two Sam domains own a canonical Sam fold made up of a five-helix bundle with a short α3 helix and form a dimer with binding affinity in the low micromolar range [15]. Both NMR [16,17] and X-ray crystallography [18] have clarified the structural details of the EphA2–Sam/Ship2–Sam association; the two Sam domains bind following the End Helix (EH)/Mid Loop (ML) model that is representative of several Sam–Sam interactions (Figure 1).

The interaction between Ship2–Sam and EphA2–Sam is possibly connected to pro-oncogenic outcomes, as Ship2 downregulates ligand-induced EphA2 receptor activation (i.e., anti-oncogenic signalling) while enhancing a ligand-independent pro-migratory activity (i.e., pro-oncogenic signalling) [16]. On the other side, it has also been demonstrated that EphA2–Sam lowers receptor oligomerization and kinase activation [19,20] and might favour pro-oncogenic monomeric or low-oligomeric receptor forms, possibly through interaction with the kinase domain or other Sam partners like Ship2 [19,21].

To further understand the correlation between the EphA2–Sam/Ship2–Sam interaction and cancer onset and progression, we here present a computational approach focused on cancer-related somatic mutations affecting the EH and ML interfaces in EphA2–Sam and Ship2–Sam, respectively. Such missense mutations were retrieved from the COSMIC (Catalogue of Somatic Mutations in Cancer) database [22], and primary sequence analyses were conducted with bioinformatic tools in EXPASY [23] to point out variations in chemical–physical properties induced by mutations. Thus, 3D structural models of mutant Sam domains were predicted with AlphaFold2 (AF2) [24] and analysed with UCSF Chimera [25] and MolMol [26]. FoldX [27,28], Hotmusic [29,30], PopMuSiC (Prediction of Protein Mutant Stability Changes) [31], Maestro (Multi Agent Stability Prediction upon point mutations) [32], and INPS-3D (Impact of Non synonymous variations on Protein Stability-3D) [33] software were employed to predict stabilizing and destabilizing mutations. Stabilizing and destabilizing factors characterizing a few mutants were further investigated by molecular dynamics simulations. Finally, the HADDOCK (High Ambiguity Driven protein-protein DOCKing) Refinement Interface [34] was employed to analyse if and how certain mutations could affect the binding of EphA2–Sam to Ship2–Sam. Although experimental validation will be needed to undoubtedly prove the in silico gained knowledge, our study provides a robust protocol, employing cutting-edge computational instruments to select those cancer-related mutations whose pathological outcomes might be linked to the modulation of EphA2 receptor endocytosis by interfering with heterotypic EphA2–Sam/Ship2–Sam associations and on which to centre subsequent experimental analyses.

## 2. Results and Discussion

**Figure 2:**
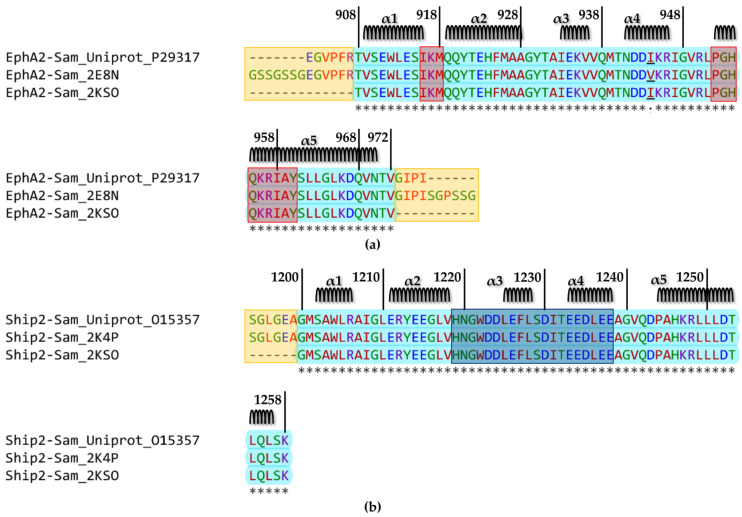
(**a**) Alignment of the human native EphA2–Sam sequence (UniProtKB [35] code P29317) with those corresponding to the NMR structures of the isolated domain (pdb entry 2E8N) and in complex with Ship2–Sam (pdb entry 2KSO [16]). The alignment was generated with the Clustal Omega multiple sequence alignment program [36] (https://www.ebi.ac.uk/Tools/msa/clustalo/ access date 4 June 2023). Cyan is used to highlight the amino acids shared by all three sequences (residue range T908–V972) except residue 944 that is underlined and mutated to valine in the sequence of the NMR structure pdb entry 2E8N. The secondary structure elements are reported on the top, and the ranges for α-helices were defined by MolMol [26] analysis of the first conformer in the 2E8N ensemble of structures (i.e., α1 V909–I916, α2 Q919–A928, α3 I933–V936, α4 N941–R946 and α5 P952–N970). The EH interface (residues I916–M918 and P952–Y960) is indicated by red rectangles. The flexible N- and C-terminal tails in the 2E8N structure are highlighted by yellow rectangles. (**b**) Alignment of the human Ship2–Sam sequence retrieved from UniProtKB (code O15357) and those of the NMR structures of the isolated domain (pdb entry 2K4P [17]) and in complex with EphA2 (pdb entry 2KSO [16]). The alignment was generated with the Clustal Omega multiple sequence alignment program [36] (https://www.ebi.ac.uk/Tools/msa/clustalo/ access date 4 June 2023). The residues with negatively charged side chains (D, E) are indicated in blue. The residues with both polar aliphatic and aromatic side chains (H, N, Q, S, T, Y) and G are indicated in green. The residues with both apolar aliphatic and aromatic side chains (A, F, I, L, M, P, V, W) are indicated in red.The residues with positively charged side chains (K, R) are indicated in violet; asterisks indicate fully conserved residues. The amino acids shared by all three sequences (G1200–K1258) are coloured in cyan. Secondary structure elements are indicated on the top, and were defined based on MolMol [26] inspection of the first NMR conformer in pdb entry 2K4P (i.e., α1 S1202–R1206, α2 E1211–V1218, α3 L1225–L1228, α4 E1233–E1238 and α5 P1244–L1256). The ML interface (residues H1219–E1238) is highlighted by a darker shaded rectangle. The yellow rectangle indicates the flexible N-tail in the 2K4P [17] entry.

The Sam domain (Figure 2a) encompasses residues V904 to Q968 within the sequence of human EphA2 (UniprotKB [35] entry P29317). The structure of this Sam domain has been solved by NMR techniques (pdb entry 2E8N) and contains the mutation I944V with respect to the wild-type sequence. The structure of the human EphA2–Sam domain in complex with human Ship2–Sam has also been obtained by NMR techniques (pdb entry 2KSO [16]) (Figure 1 and Figure 2a). 

NMR studies have also been conducted for human Ship2–Sam (UniprotKB [35] entry O15357 residues from L1196 to K1258) as an isolated domain (pdb entry 2K4P [17]) (Figure 2b).

As briefly mentioned in the Introduction, the EphA2–Sam/Ship2–Sam complex adopts the EH/ML interaction structural topology (Figure 1) [16,17]. EphA2–Sam forms the EH surface, which is made up of residues from the C-terminal α5 helix and the adjacent α1α2 loop (Figure 1 and Figure 2a), and the Ship2–Sam central region forms the ML interface, which is made up mostly of the C-terminal part of α2, α3, α4 helices, and interhelical loops (Figure 1 and Figure 2b) [37]. Electrostatic contacts highly contribute to complex formation as the EphA2–Sam EH surface is positively charged, whereas the Ship2–Sam ML interface is negatively charged (Figure 1 and Figure 2) [16,17,18]. 

Within this study, we employed, for comparison purposes, when analysing isolated Sam domains, the best conformers of NMR structures (i.e., pdb entry 2E8N and 2K4P [17] for EphA2–Sam and Ship2–Sam, respectively), after deletion of the flexible N- and C-terminal regions (Figure 2a,b). For analysis of intermolecular interactions between EphA2–Sam and Ship2–Sam, the pdb entry 2KSO [16] was instead employed as a reference structure.

### 2.1. Cancer-Related Mutations in EphA2–Sam and Ship2–Sam

COSMIC is a great resource for the scientific community involved in cancer-related projects as it brings together the existing knowledge concerning somatic mutations and their outcomes within the vast array of human cancers [22]. We searched for cancer-related missense mutations, leading to punctual amino acid variations within the sequences of Sam domains from EphA2 and Ship2 through the COSMIC v98 database [22], and the results are summarized in Table 1 and Table 2 for EphA2–Sam and Ship2–Sam, respectively. 

Residues crucial for structure and function are generally located in conserved positions within a protein family, and related mutations are often associated with pathological outcomes. The majority of missense mutations linked to pathological conditions are usually connected to structural changes and/or decreased protein stability and affect catalytic activities and/or binding properties [51].

**Figure 3:**
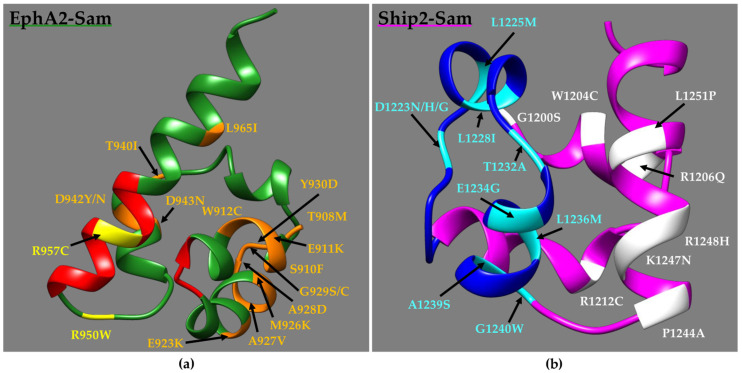
(**a**) NMR structure of EphA2–Sam (dark green) (first conformer -residues T908–V972-, pdb entry 2E8N without flexible N- and C-terminal tails) in a ribbon representation. The EH interface (residues I916–M918 and P952–Y960) is highlighted in red, while diverse mutations are coloured in orange and yellow if positioned far away from or inside/close to the EH, respectively. Mutations V904G and R907C/S are not shown as located in the N-terminal flexible tail. (**b**) NMR Structure of Ship2–Sam (magenta) (first conformer -residues G1200–K1258-, pdb entry 2K4P [17] after removal of the flexible N-tail) in a ribbon representation. The ML interface (residues H1239–E1238) is coloured in blue; mutations are highlighted in white and cyan if positioned far away from or inside/close to the ML, respectively. Mutation E1198K is not shown as included in the N-terminal disordered tail.

Analyses of NMR structures of Ship2–Sam and EphA2–Sam with the Consurf web server (https://consurf.tau.ac.il/consurf_index.php access date, 19 June 2023) [52,53] (Tables S1 and S2), which is widely implemented to establish the evolutionary conservation degree of amino acid positions in proteins, indicate that not all the EphA2–Sam (Figure 3a and Table 1 and Table S1) and Ship2–Sam (Figure 3b and Table 2 and Table S2) cancer-related missense mutations concern highly conserved amino acids. Indeed, for EphA2–Sam (Table S1), only 8/17 mutations in the folded domain (residues 908–972) affect rather conserved positions (i.e., conservation scores higher than 5).

For Ship2–Sam (Table S2), the conservation degree for mutated residues within the folded domain (residues G1200–K1258) appears larger, with 15/18 mutations affecting conserved positions (ConSurf scores higher than 5 [52]). 

For EphA2–Sam, two point mutations (i.e., R950W and R957C (Figure 3a)) are localized within or close to the EH interface that is responsible for binding Ship2–Sam, thus letting us speculate they might influence Sam–Sam association. Indeed, previous studies conducted by employing mouse sequences indicate that the R957C EphA2–Sam mutant is unable to bind Ship2–Sam due to the loss of crucial electrostatic interactions provided by the R957 side chain [18]. Another two mutations that are positioned outside the EH interface (i.e., W912C and D943N (Figure 3a)) destabilize the EphA2–Sam fold, and these mutants are expressed as inclusion bodies [18]. The mutation T940I (Figure 3a) has instead also been associated with cataracts and loss of cell migration ability. Nevertheless, the expression of GST-fused T940I EphA2–Sam domain decreases the solubility of EphA2–Sam due, possibly, to misfolding or aggregation [54]. Moreover, the residue R950 (Figure 3a) has been previously investigated through mutagenesis studies, and its substitution to Threonine results in increased binding to Ship2–Sam [16].

For Ship2–Sam, 10 mutations are positioned inside or in close proximity to the ML binding surface for EphA2 (Figure 3b).

It is worth noting that several mutations affect D1223 (Figure 3b), which represents a residue providing interactions crucial for EphA2–Sam binding; in fact, it has previously been reported that the double-mutant D1223A/D1224A loses the ability to interact with EphA2–Sam [16]. 

### 2.2. Investigating EphA2–Sam and Ship2–Sam Mutant Stability by In Silico Approaches

#### 2.2.1. Predictions Based on Amino Acid Sequences

The Protparam tool in EXPASY [23,55] was employed to predict possible changes in the chemical–physical properties of EphA2–Sam (Table S3) and Ship2–Sam (Table S4) induced by cancer-related mutations. For EphA2, we analysed data for both the wild-type sequence and the I944V mutant for which the NMR structure is available (Figure 2a). 

The grand average of the hydropathy (GRAVY) value for a protein is obtained by summing hydropathy values of the single amino acid residues and then dividing the results by the total number of residues [55]. Negative GRAVY values indicate hydrophilic proteins, and positive values point out hydrophobic ones [56]. Given all negative values, EphA2–Sam (Table S3) and Ship2–Sam (Table S4) sequences appear as hydrophilic. For both EphA2–Sam and Ship2–Sam, the differences in the GRAVY values between mutant and wild-type sequences are rather small. EphA2–Sam mutations with the largest effect on GRAVY are M926K, which should increase the hydrophilicity, and R957C, which is positioned in the EH interface (Figure 3a) and should produce, instead, an increase in hydrophobicity (Table S3). 

For Ship2–Sam (Table S4), the biggest effects are observed for the R1212C (Figure 3b) and L1251P (Figure 3b) mutants that, however, are not positioned inside the Ship2–Sam ML interface (Table S4).

Concerning the pI (Isoelectric point) values, the largest changes occur in EphA2–Sam (Table S3) upon inserting the E911K, E923K, R950W, and R957C mutations, while no large variations can be associated with Ship2–Sam mutations (Table S4). 

The instability index (Tables S3 and S4) can be employed to formulate hypotheses on how the mutation can affect Sam domain stability in vitro and is based on the occurrence within the sequence of precise dipeptide motives [55,57]. Instability index values smaller than 40 point out a stable protein [55]. Higher stability is associated with EphA2–Sam with respect to Ship2–Sam. Based on this prediction, the most destabilizing effect on EphA2–Sam should be associated with the W912C (Figure 3a) mutation that is positioned outside the EH interaction interface, in agreement with experimental data, demonstrating that this sequence is expressed as inclusion bodies like the D943N mutant (Figure 3a), for which a large destabilizing effect can be seen (Table S3) [18]; the largest stabilizing effect is instead observed for the R957C mutation (Figure 3a), which is positioned inside the EH interaction site (Table S3). 

Concerning Ship2–Sam (Table S4 and Figure 3b), the most destabilizing mutations should be R1212C (Figure 3b), which is positioned outside the ML interface. Instead, within the ML binding site, mutations A1239S (Figure 3b) and E1234G (Figure 3b) are those associated with the highest destabilizing and stabilizing effects, respectively, although differences with wild-type protein are relatively small (Table S4).

For EphA2–Sam, including the I944V mutation and its cancer-related mutants, instability indexes tend generally to increase, but the trend is very similar to what was observed for the variants containing I944. This is not true for the I944V-D943N mutant, for which a decrease in the instability index can be observed, likely due to the mutation of two consecutive residues, leading to novel dipeptide motives with different weight values of instability [57] (Table S3). 

#### 2.2.2. Three-Dimensional (3D) Structural Models Generation and Analysis

Missense mutations connected to pathological conditions can either generate protein destabilization, leading to unfolding, or influence protein interaction networks.

Thus, to gain further insights into potential effects due to cancer-related mutations that, at the molecular level, could affect EphA2–Sam and Ship2–Sam structure stabilities and/or interaction properties, we built and analysed 3D structure models (Figures S1–S3). We focused our analyses on those mutations localized inside or very close to the EH and ML reciprocal interaction interfaces of EphA2–Sam and Ship2–Sam, respectively, to gain a better understanding of their potential outcomes on the heterotypic Sam/Sam association and, consequently, receptor endocytosis process.

Three-dimensional (3D) models of mutant proteins were predicted with AlphaFold2 (AF2) (Figures S1–S3) [24,58]. As pointed out before (Figure 2a), the NMR structure of the isolated EphA2–Sam domain (pdb entry 2E8N) contains the I944V mutation with respect to the natural sequence, so we predicted models of EphA2–Sam cancer-related mutants with and without the I944V mutation to allow for comparison with the experimental structure (Figures S1 and S2). AlphaFold2 reference models were predicted as well for wild-type EphA2–Sam (Figure S1), for the I944V EphA2–Sam (Figure S2), and for native Ship2–Sam (Figure S3) to be able to accurately compare the different features of native and mutated Sam domains by employing structures provided with similar precisions as generated with identical protocols. 

**Figure 4:**
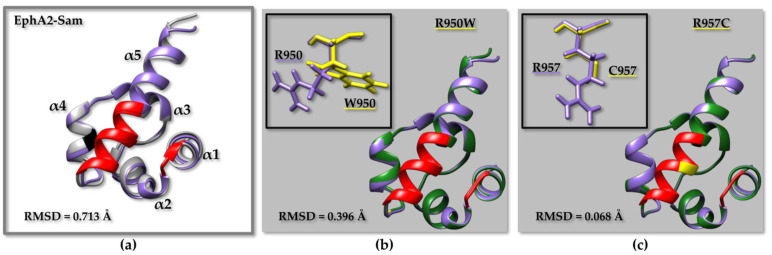
(**a**) Superimposition on the backbone atoms of EphA2–Sam NMR structure (first conformer, pdb entry 2E8N after removal of the flexible N- and C-terminal tails, residue range T908 to V972) (light grey) and the EphA2–Sam wild-type AF2 [24,58] model (violet). The backbone of residue Ile 944 is highlighted in black. (**b**,**c**) Overlays on the backbone atoms (residue range T908 to V972) of AF2 models of EphA2–Sam wild-type (violet) and cancer related mutants (dark green): (**b**) R950W, (**c**) R957C. The backbone of mutated residues is coloured yellow on the ribbon representations. The side chains of native and mutated residues are shown in violet and yellow, respectively in the upper left inserts (**b**,**c**). The EH surface in all panels is coloured in red (residues I916–M918 and P952–Y960), RMSD values are also indicated in each panel.

**Figure 5:**
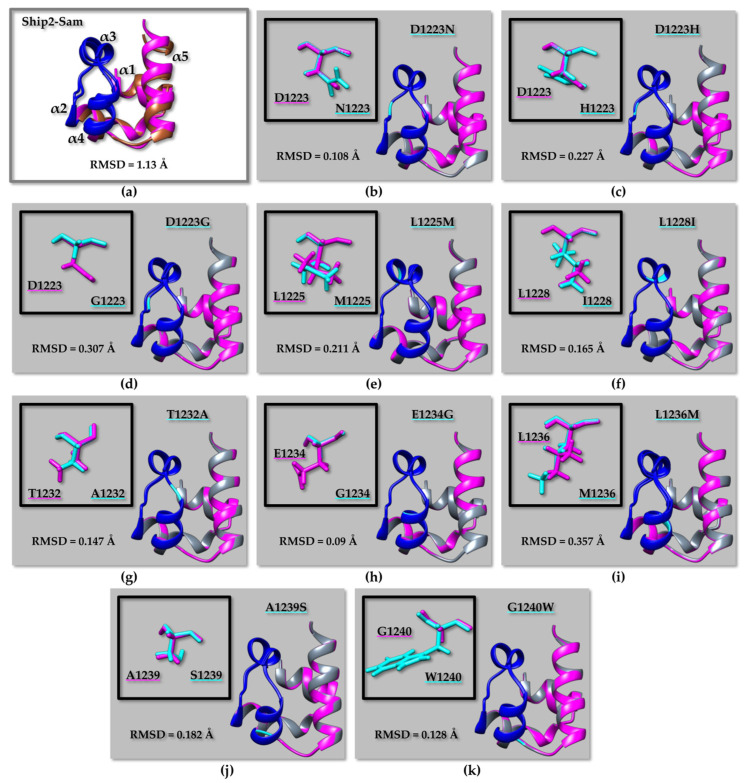
(**a**) Superimposition on the backbone atoms of Ship2–Sam NMR structure (first conformer, pdb entry 2K4P [17] without the flexible N-tail, residue range G1200–K1258) (brown) and corresponding AF2 [24,58] model (magenta). (**b**–**k**) Overlays on the backbone atoms of AF2 models of Ship2–Sam (magenta) and cancer-related mutants (grey): (**b**) D1223N, (**c**) D1223H, (**d**) D1223G, (**e**) L1225M, (**f**) L1228I, (**g**) T1232A, (**h**) E1234G, (**i**) L1236M, (**j**) A1239S, (**k**) G1240W. Only mutations in or close to the ML interface are shown. The ML interface (residues H1239–E1238) is coloured in blue in all panels. The backbone of mutated residues is coloured in cyan, whereas side chains of native and mutated amino acids are shown in magenta and cyan, respectively, in the upper left inserts. RMSD values are indicated in each panel.

Indeed, RMSD (root mean square deviation) analyses (Figure 4, Figure 5, and Figure S4; Table S5) indicate that the largest differences can be encountered when comparing experimental NMR structures with AF2 models, although the two structures remain practically identical concerning the secondary structure elements, while small differences are encountered in the most disordered regions (Figure 4a, Figure 5a and Figure S4a; Table S5). The observed differences might be due to the dissimilar structure refinement protocols of AF2 models compared to the experimental NMR structures. For example, the Ship2–Sam structure (pdb code 2K4P [17]) was calculated with the software CYANA 2.1, which performs simulated annealing in the torsion angle space [59], without further energy minimization. 

Next, we compared AF2 models for wild-type and mutated Sam domains without revealing major differences (Table S5). However, it needs to be pointed out that, according to previous studies, AF2 is unable to precisely predict the structural outcomes of missense mutations by employing, as input, the sequence of a mutated protein as there will be a bias towards wild-type or homologous sequences [60,61]. In fact, AF2 cannot predict very large structural changes or unfolding induced by a mutation while generally providing something like the native structure as an output model [62]. With this in mind, we employed the AF2 models of mutant Sam domains just to draw some preliminary structural insights that, as will be described later, will be further checked by diverse in silico tools and molecular dynamics simulations. 

When superimposing the AF2 models for WT EphA2–Sam and the R950W (Figure 4b) or R957C (Figure 4c) mutants, a larger difference between the structures can be observed for R950W (Figure 4b) (Table S5). The same is not true if considering the AF2 model of the I944V EphA2–Sam domain as it can be superimposed to the atomic coordinates of I944V-R950W (Figure S4b) and I944V-R957C (Figure S4c) mutants, producing lower RMSD values for the mutant provided with the R950W substitution (Table S5). This outcome could be linked to the larger sidechain of Ile944 with respect to Val944. It can be supposed that a slightly larger structure perturbation is needed to place a Trp in position 950 when position 944 contains the larger side chain of Ile instead of Val (Figure 4 and Figure S4). In fact, residue 944 is located on the α4 helix rather close to residue 950, which is instead positioned in the α4α5 loop. 

For Ship2–Sam (Figure 5 and Table S5), RMSD analyses predict that the L1236M mutant should be affected by a certain structural variation with respect to the wild type when considering overlays on the backbone of either all residues or the ML region (Figure 5i and Table S5) followed by the D1223G mutant (Figure 5d and Table S5). The residue L1236 is located inside the protein core (See Table S2), and it is to be expected that mutations affecting core residues might have the largest impact on the structure. 

Next, to gain further insights into the stabilizing and destabilizing factors associated with cancer-related missense mutations affecting the EH and ML interfaces, we predicted variations in the Gibbs free energy (ΔΔG) induced by mutations. Previous studies pointed out that to generate more accurate predictions, it is better to compare results from diverse tools [51,63,64]. Thus, we implemented the following structure-based predictors: FoldX [27,28], PopMuSiC (https://soft.dezyme.com/query/create/pop, access date 16 June 2023) [31], Maestro (Multi Agent Stability Prediction upon point mutations) (https://pbwww.services.came.sbg.ac.at/maestro/web, access date 25 June 2023) [32], INPS-3D (Impact of Non synonymous variations on Protein Stability-3D) (https://inpsmd.biocomp.unibo.it/inpsSuite/default/index3D, access date 25 June 2023) [33]. These predictors, except INPS-3D [33], evaluate the folding free energy variation induced by the mutation (ΔΔG) by computing the difference between the folding free energy of the mutant and wild type (ΔGfmut-ΔGfwt), and, accordingly, stabilizing mutations are associated with a negative ΔΔG sign.

Instead, INPS-3D estimates the difference in the Gibbs free energy change between wild-type and mutant proteins and labels destabilizing mutations with a negative sign [65]. The signs of INPS scores were inverted to avoid confusion among diverse predictors (Table 3). 

ΔΔG predictions, to date, cannot be considered perfect, and, in general, the results are unreliable when the mutation causes a ΔΔG within the interval ±0.5 kcal/mol [64]. As concerning FoldX-generated ΔΔG values, those larger than 1.6 kcal/mol should be considered highly significant (2 standard deviations of the FoldX error, 99% confidence interval), but energy variations greater than 0.8 kcal/mol can still be considered reliable (1 standard deviation, 95% confidence interval) [66]. Within our analyses (Table 3), predicted ΔΔG values were considered unreliable if falling within the ±0.5 kcal/mol interval for all predictors except FoldX, for which a ±0.8 kcal/mol range was taken into account. Nevertheless, we also classified as destabilizing those mutations with ΔΔG > 1 kcal/mole and stabilizing those with ΔΔG < −1 kcal/mole (Table 3). 

For EphA2–Sam, more reliable predictions could be obtained for the R957C mutant that, however, presents small ΔΔG variations and should be considered as a neutral mutation, not expected to largely influence the EphA2–Sam structure. Indeed, R957 has a solvent exposure higher than 30% (Table S1), and, as already demonstrated by experimental studies, its mutation to cysteine negatively affects binding between Ship2–Sam and EphA2–Sam [18]. 

Concerning Ship2–Sam (Table 3), agreement between at least 3/4 predictors points out that L1228I (Figure 5f) and T1232A (Figure 5g) should be linked to destabilizing effects and, similarly, 2/4 predictors associate destabilizing outcomes to L1236M (Figure 5i). Interestingly, L1228 and L1236 have poor solvent exposure (Table S2), and the mutations of these residues are indeed expected to generate a perturbation in the protein core, thus likely affecting the overall fold. 

A previous study pointed out that mutations associated with pathological conditions leading to small |ΔΔG| ≤ 1 are usually positioned at high-solvent-exposed loci of the protein structure and are likely to hamper protein–protein interactions without significatively altering the overall protein structure [67]. Analyses of thermodynamic stability changes (Table 3) show that, mainly, mutations affecting residues with a solvent exposure of at least 30% in EphA2–Sam (Table S1) and Ship2–Sam (Table S2) will either produce non-reliable predictions or have a neutral effect on the overall structure. This is the case of the Ship2–Sam residue D1223, which also presents a high conservation score (Table S2 and Table 3) and is known to play a role in the association with EphA2–Sam [16,17]. All cancer-related mutations involving D1223: D1223N (Figure 5b), D1223H (Figure 5c), and D1223G (Figure 5d), by interfering with electrostatic interactions at the ML(Ship2–Sam)/EH(EphA2–Sam) interface, might negatively modulate Ship2–Sam/EphA2–Sam complex formation. Similarly, mutation of the more exposed residue E1234 (Table S2) to Gly (Figure 5h) is not predicted to induce a large structural variation (Table 3) but could interfere with electrostatic interactions, again influencing the binding of Ship2–Sam to EphA2–Sam. Finally, predictions for G1240W mutation (Table 3) are rather in disagreement, as 2/4 predictors indicate destabilizing effects, but, in the case of FoldX, the prediction is associated with a large Van Der Waals clash penalty [27,28].

A further analysis was conducted with the tool HotMusic (https://soft.dezyme.com/query/create/hot, access date 26 June 2023) [29,30] that predicts variations in melting temperature due to point mutations (Table S6). For the D1223H and A1239S mutations in Ship2–Sam, Hotmusic [29,30] predicts a decrease in thermal stabilities for most EphA2–Sam and Ship2–Sam mutants (Table S6). 

To better understand the effects of cancer-related missense mutations on EphA2–Sam and Ship2–Sam structures, we performed molecular dynamics simulations of a few mutants. 

Molecular dynamics was already implemented to study the effect of the melanoma-related mutation L920F in EphA4-Sam, revealing that this mutation might cause a conformational change in EphA4-Sam, thus affecting its oligomerization state [68]. A recent study analysed the possible outcomes induced by several non-small-cell lung cancer (NSCLC)-related missense mutations on the structures of the ephrinA2 receptor binding domain, EphA3 ligand binding, and kinase domains, and the EphA7, EphB1, and EphB4 kinase domains through MD simulations [69]. 

In detail, we chose to more deeply investigate the Ship2–Sam mutant G1240W, for which ΔΔG predictors were producing ambiguous results; D1223G and D1223H, which could influence binding between Ship2–Sam and EphA2–Sam, as indicated by experimental evidence [16]; A1239S, since, although A1239 is not associated with a very large solvent exposure (Table S2), is a mutation that according to ¾ predictors should not induce large structural perturbations; and T1232A, for which mainly a destabilizing effect is predicted. Interestingly, the selected Ship2–Sam mutants are among those provided with the high Consurf scores (Table S2) that could, consequently, have a certain impact on Ship2–Sam structure and interaction properties [52,53].

For EphA2–Sam, as it was already reported a role of the R957C mutation in deeply influencing binding to Ship2–Sam, we focused on the R950W mutation, trying to gain some insights on the relationship between structural variations and pathological conditions, also considering that all ΔΔG predictors (Table 3) failed to produce reliable results.

### 2.3. Molecular Dynamics

**Figure 6:**
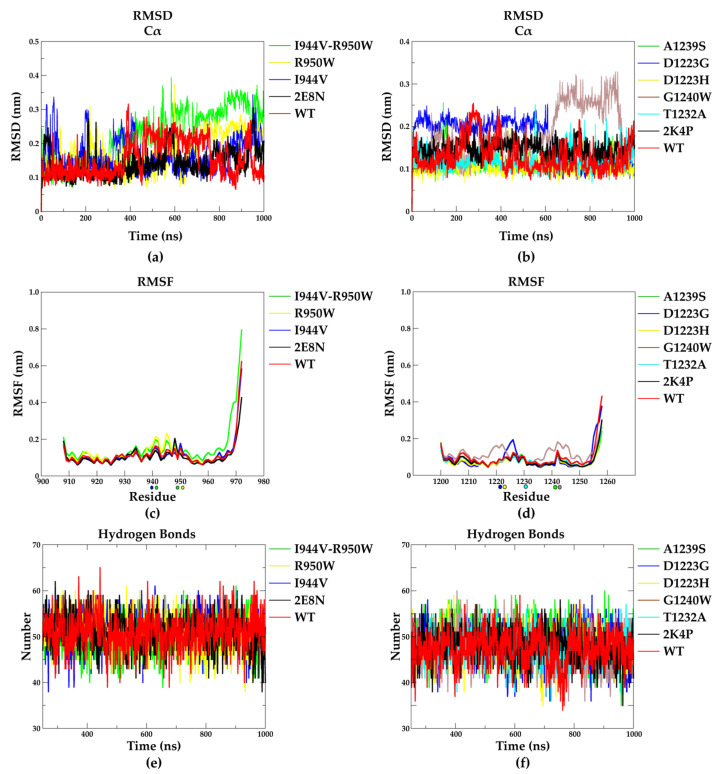
Molecular dynamics analyses. (**a**,**b**) Cα RMSD vs. MD simulation time. (**c**,**d**) RMSF plots of all residues. Mutated residues are highlighted with spheres on the *x* axes. (**e**,**f**) Number of hydrogen bonds along MD simulations. Data are shown for simulations started from either NMR structures (2E8N and 2K4P) and AF2 models of wild-type (WT) and mutant EphA2–Sam (**a**,**c**,**e**) and Ship2–Sam forms (**b**,**d**,**f**).

To investigate the effect of the mutations on the global structure and dynamics of EphA2–Sam and Ship2–Sam, the AF2 models and the experimental NMR structures (pdb entries: 2E8N and 2K4P [17], for EphA2–Sam and Ship2–Sam, respectively) were subjected to molecular dynamics (MD) simulations for 1 microsecond in explicit solvent and counter-ions. Along the simulation trajectories, the Cα atoms’ root mean square deviation (RMSD) profiles suggest that no significant variations from the initial conformations occur (Figure 6a,b and Table S7).

The trajectories relative to the AF2 models of wild-type EphA2–Sam and Ship2–Sam domains and those started from the experimentally solved NMR structures (Figure 6a,b red and black profiles) all show very low and comparable mean and standard deviation values of RMSD (Table S7), while I944V–R950W EphA2–Sam and G1240W Ship2–Sam mutants show slightly higher deviations (Figure 6a,b and Table S7). Consistently, an inspection of the root mean square fluctuation (RMSF) profiles of the EphA2–Sam mutants (Figure 6c) indicates that the R950W is the model with the highest flexible profile, followed by the I944V–R950W, particularly in the region close to mutations (T940–R950) (Figure 6c). Among the Ship2–Sam mutants, the G1240W and D1223G variants present the largest increase in flexibility (Figure 6d), but although these two mutations affect the Ship2–Sam domain to a larger extent than the others, the differences are not so relevant (Figure 6d). We also monitored the hydrogen bond network within each domain along the relative MD simulations, and the obtained data are comparable for all the EphA2–Sam and Ship2–Sam mutants (Figure 6e for EphA2–Sam and 6f for Ship2–Sam and Table S7). The representative states of each EphA2–Sam and Ship2–Sam variant extracted from the trajectories based on RMSD criteria are shown in Supplementary Figure S5. Despite a small but expected increase in local flexibility shown by the mutated domains, globally, MD analyses indicate that the considered mutations do not strongly affect the conformation and dynamics of EphA2–Sam and Ship2–Sam in their apo forms. The trajectory data support the idea that the considered residues might have a crucial role in molecular recognition processes involving EphA2–Sam or Ship2–Sam, likely by differentially influencing the Sam interaction interfaces.

### 2.4. Effect of Point Mutations on the Structure and Affinity of the EphA2–Sam/Ship2–Sam Complex

To predict how cancer-related mutations could affect the association between EphA2–Sam and Ship2–Sam, we first employed the Haddock refinement interface [34] to evaluate structural changes in the binding topology, whereas dissociation constant values were estimated with the Prodigy webserver [70]. Further analyses of residues at the Sam–Sam interface and intermolecular contacts were carried out with Ligplot+ [71,72]. 

#### 2.4.1. Computational Method Validation

As mentioned above, the structural features of the human EphA2–Sam/Ship2–Sam complex have been studied in detail using NMR techniques [16,17]. This Sam–Sam interaction is mostly driven by intermolecular contacts in between the positively charged residues from the EH surface in EphA2–Sam and negatively charged ones from the ML surface in Ship2–Sam (Figure 1) [16,17]. The diverse conformational families and all intermolecular interactions characterizing the rather dynamic EphA2–Sam/Ship2–Sam complex have been previously discussed [16,17]. However, to be able to carry out a strict comparison of data deriving from the same in silico procedure, we also applied our Haddock, Prodigy, and Ligplot+ analyses (see Materials and Methods for further details) to the EphA2–Sam/Ship2–Sam native complex (Tables S8 and S9).

Starting from a few EphA2–Sam/Ship2–Sam NMR conformers, the Haddock refinement interface generated 99 optimized structures. Refinements with Haddock were performed for histidine residues in an uncharged state to be consistent with previous NMR structural calculations [16]. According to our clusterization protocol (see Materials and Methods for details), the resultant 99 refined structures could be subdivided into two clusters, and the best cluster (in terms of Haddock score) also corresponded to the most populated one (Table S8) [34,73,74,75]. Intermolecular contacts in the EphA2–Sam/Ship2–Sam complex are summarized in Figure S6 and correspond to all the previously reported characteristic interactions of this Sam–Sam association [16,17]. Moreover, the dissociation constant predicted by Prodigy [70] is not too discordant from the experimental value when considering structures belonging to the first cluster (Table S9). It also needs to be pointed out that K_D_ measurements may be dependent on experimental conditions, and, in fact, studies conducted in another laboratory reported for the EphA2–Sam/Ship2–Sam complex a K_D_ value equal to 0.75 ± 0.12 µM evaluated in PBS at pH 7.7 [17].

Previous work revealed that mutation of the EphA2–Sam residue R950 in threonine should increase the binding affinity for Ship2–Sam (Table S9) by decreasing electrostatic repulsion between R950 (EphA2–Sam) and H1219 (Ship2–Sam) (Figure S6) [16]. Thus, to further test our in silico approach, we modelled the R950T EphA2–Sam/Ship2–Sam interaction (Figure S7). Prodigy [70] predicted K_D_ for the R950T EphA2–Sam/Ship2–Sam association appears to be in good agreement with experimental data, although the Haddock scores of the mutant and wild-type complex are not too dissimilar (Table S9). However, when considering structures provided with positively charged histidines, again, differences in K_D_ values and Haddock scores between the native and R950T mutated complexes remain small, although confirming a certain improvement in binding affinity (Table S10), as seen experimentally. 

Inside the EphA2–Sam EH interface, residue K956 appears to be a crucial provider of intermolecular contacts [16,17], and its mutation to aspartic acid dramatically decreases Ship2–Sam/EphA2–Sam association [16]. In fact, K956 is positioned on the EphA2–Sam α5 helix (Figure S6) and is able to interact with several negatively charged residues in the Ship2–Sam ML area, such as E1234, D1235, and E1238 (Figure S6). 

We modelled and optimized the K956D EphA2–Sam/Ship2–Sam association with Haddock (Figure S8) [75]. Due to the loss of interactions induced by inverting the K956 positive charge in the negative charge of an aspartic and the repulsions deriving from identically charged residues facing each other at the Sam/Sam binding surface, a marked increase (i.e., a worsening) in the Haddock scores is evidenced (Table S9). Unexpectedly, and contrarily to Haddock results [34], Prodigy [70] predicts a dissociation constant value for the K956D EphA2–Sam/Ship2–Sam complex in the same order of magnitude of the wild-type interaction. Thus, Prodigy fails to predict the large decrease in binding affinity that was experimentally observed (Table S9). 

In summary, this computational protocol let us speculate that Haddock scores can provide a better indication of the decrease in binding affinity that a mutation can induce with respect to the Prodigy prediction. The method also seems to work better when large differences in the interaction affinity between mutant and wild-type variants are induced by a certain mutation (Table S9). 

Next, similar computational analyses were conducted on the cancer-related mutated EphA2–Sam/Ship2–Sam complexes (Tables S11 and S12).

#### 2.4.2. R950W EphA2–Sam/Ship2–Sam Interaction

**Figure 7:**
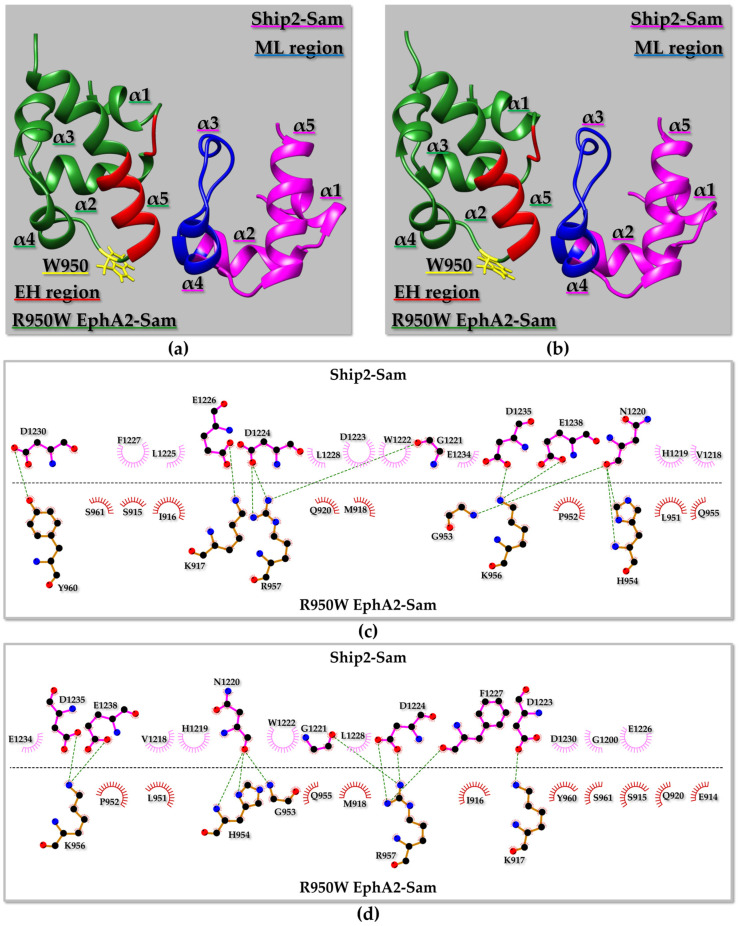
(**a**,**b**) Ribbon representation of the R950W EphA2–Sam/Ship2–Sam complex: the best structure from the best Haddock [34] cluster is shown in (**a**), whereas the best structure from the most populated cluster is shown in (**b**). The mutated residue is highlighted in yellow, and its side chain is shown as well; the EH and ML interaction interfaces in R950W EphA2–Sam, and Ship2–Sam are coloured in red and blue, respectively. (**c**) 2D diagram of intermolecular interactions generated by LigPlot+ [71,72] analysis of the binding interface in the R950W EphA2–Sam/Ship2–Sam complex shown in panel (**a**). (**d**) 2D diagram of intermolecular contacts identified by LigPlot+ [71,72] for the R950W EphA2–Sam/Ship2–Sam complex shown in panel (**b**). (**c**,**d**) Carbon, Nitrogen, and Oxygen atoms are indicated by black, blue, and red spheres, respectively. H-bonds are highlighted with green dashed lines. R950W EphA2–Sam and Ship2–Sam residues involved in non-bonded interactions are labeled and represented by red and pink crescents with bristles.

The residue R950 is positioned close to the EphA2–Sam EH interface (Figure 7a,b) and has a rather high solvent exposure (Table S1). In the wild-type complex, residue R950 from EphA2–Sam is involved in intermolecular interactions with H1219 from Ship2–Sam (Figure S6); as both R and H are positively charged, repulsion can occur at the lowest pH between their side chains. Indeed, previous studies demonstrated that the R950T mutation induces an increase in the affinity of EphA2–Sam for Ship2–Sam, as explained before [16]. 

All 99 optimized Haddock structures could be grouped into three clusters of conformationally related families, where the best cluster (i.e., first cluster) in terms of lowest Haddock score did not correspond to the most populated one (i.e., second cluster) (Table S12 and Figure 7a,b). Intermolecular interactions characterizing the best Haddock structures from the first and second clusters of the R950W mutant complex can be seen in Figure 7c,d. The pattern of intermolecular contacts characterizing the best structure from the first cluster differs slightly from that in the best structure from the second cluster (Figure 7c,d). Interestingly, the canonical intermolecular H-bond characterizing EH-ML Sam–Sam complexes [76], which involves a glycine at the N-terminus of the α5 helix on the EH surface and a residue at the C-terminal side of the α2 helix on the ML interface (i.e., EphA2–Sam G953 _N_H/Ship2–Sam N1220 O), is preserved in the R950W EphA2–Sam/Ship2–Sam complex (Figure 7c,d).

Our computational approach points out that the R950W mutation should not dramatically influence the interaction between EphA2–Sam and Ship2–Sam based on Haddock scores and predicted K_D_ values (Table S11). Only slightly improved Haddock scores and predicted K_D_ values are encountered if refinements are performed with charged histidine residues (Table S10) [16]. 

Similarly to that previously discussed for the R950T EphA2–Sam/Ship2–Sam complex, and keeping in mind that replacement of R950 with an uncharged residue could avoid repulsion between R950 (EphA2–Sam) and H1219 (Ship2–Sam), it is possible to hypothesize that, under certain experimental conditions and especially pH values favouring a histidine charged state, replacement of the positively charged residue R950 with the uncharged tryptophan might have a positive effect on the Sam–Sam association by decreasing electrostatic repulsions at the Sam–Sam binding interface. However, it cannot be excluded that this favourable effect of the mutation could also be accompanied by unfavourable steric and solvation effects linked to the insertion in an exposed site of the large and more hydrophobic aromatic system of Trp. As evidence for the R950T mutation, the combined Haddock and Prodigy approach appears unable to predict modest variations in the interaction affinity. Thus, based on computational data, this mutation is not expected to hamper complex formation or relevantly affect the strength of the Sam–Sam association.

#### 2.4.3. EphA2–Sam/D1223H Ship2–Sam and EphA2–Sam/D1223G Ship2–Sam Interactions

**Figure 8:**
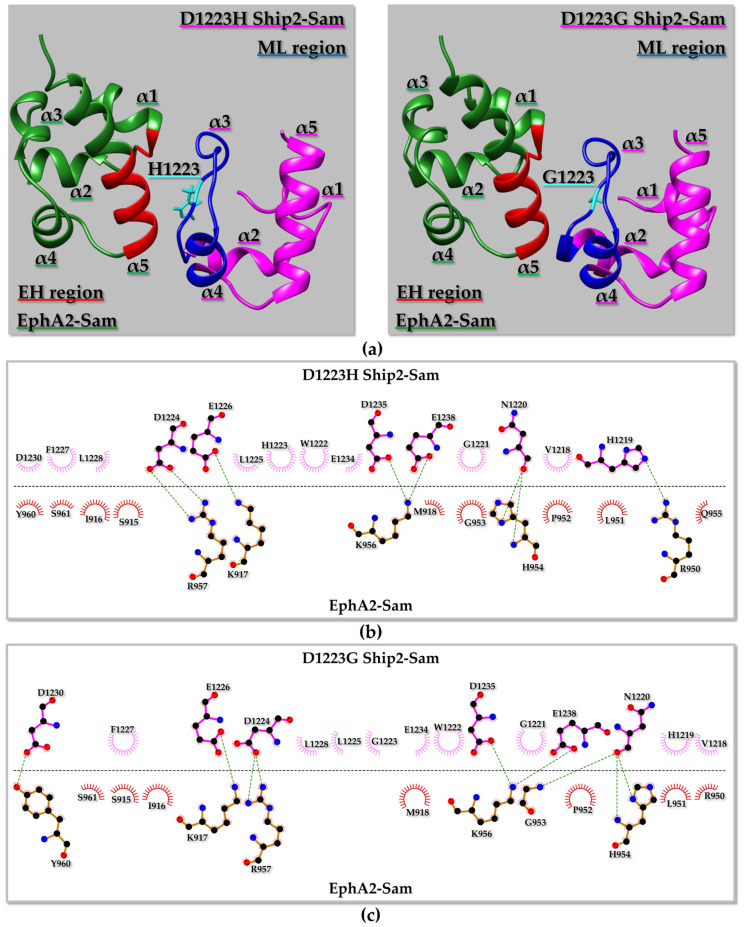
(**a**) Ribbon representation of the best structure from the best Haddock [34] cluster of the EphA2–Sam/D1223H Ship2–Sam (**Left panel**) and EphA2–Sam/D1223G Ship2–Sam (**Right panel**) complexes. The point mutation in Ship2–Sam is highlighted in cyan, and the EH and ML interaction interfaces in EphA2–Sam, and D1223H/G Ship2–Sam are coloured in red and blue, respectively. (**b,c**) 2D diagrams of intermolecular interactions generated by LigPlot+ [71,72] for the EphA2–Sam/D1223H Ship2–Sam (**b**) and EphA2–Sam/D1223G Ship2–Sam (**c**) complexes. Carbon, Nitrogen, and Oxygen atoms are indicated by black, blue, and red spheres, respectively. H-bonds are highlighted with green dashed lines. EphA2–Sam and D1223H/G Ship2–Sam residues involved in non-bonded contacts are labelled and represented by red and pink crescents with bristles, respectively.

Residue D1223 is positioned in the ML binding surface of Ship2–Sam and is also involved in several intermolecular contacts with positively charged residues on the EH interface of EphA2–Sam (Figure 8). Previous experimental studies pointed out that the double Ship2–Sam mutant D1223A/D1224A failed to interact with EphA2–Sam [16]. Interestingly, in the best Haddock structure from the first cluster, H1223 forms intermolecular contacts with several residues (Figure 8b), although, with respect to the wild-type EphA2–Sam/Ship2–Sam complex (Figure S6b), the number of intermolecular H-bonds decreases. The canonical H-bond of the EH/ML Sam–Sam complex “EphA2–Sam G953 _N_H/Ship2–Sam N1220 O” is replaced by “EphA2–Sam H954 _N_H/D1223H Ship2–Sam N1220 O” (Figure 8b), thus possibly indicating a certain distortion of the canonical EH/ML structural topology of interaction linked to the cancer-related mutation. However, based on Prodigy-predicted K_D_ values and Haddock scores, this mutation is not expected to change the affinity of Ship2–Sam for EphA2–Sam consistently at pH values that should favour histidine in the non-protonated state (Table S11). The same mutation seems to have a more, but still small, destabilizing effect at pH values that favour the charged histidine state (Table S10). This is in line with the intermolecular contacts observed between H1223 (Ship2–Sam) and positively charged residues of EphA2–Sam (such as K917 and R957) and the consequent electrostatic repulsion that could be generated by a charged H1223 (Figure 8b). 

If D1223 is instead mutated to glycine (Figure 8a right panel and Figure 8c), the electrostatic interactions provided by residue D1223 at the Sam–Sam interface are lost, the H-bond characteristic of the EH/ML Sam–Sam complexes is maintained (i.e., EphA2–Sam G953 _N_H/D1223G Ship2–Sam N1220 O), and both the Haddock scores and the K_D_ values guessed by Prodigy [70] (Table S11) allow us to speculate very small differences in the binding affinity with respect to the wild-type complex.

It is worth noting that complete abolishment of binding to EphA2–Sam induced by the Ship2–Sam D1223 mutations is not to be expected as D1223 is close to D1224 that can still supply/replace electrostatic intermolecular contacts with the positively charged EphA2–Sam EH interface to stabilize complex formation.

#### 2.4.4. EphA2–Sam/T1232A Ship2–Sam Interaction

The residue T1232 is positioned inside the Ship2–Sam ML interface and is not fully solvent exposed (Table S2). Results from the Haddock refinement interface for the corresponding mutated EphA2–Sam/Ship2–Sam complex indicate that out of 99 refined structures, 97 could be subdivided into two conformational clusters, where the number 1 corresponded to the best and most populated one (Tables S11 and S12, Figure S9). The canonical H-bond of the EH/ML Sam–Sam complex is maintained, and Haddock scores and predicted K_D_ values are very similar to those obtained for the native complex (Table S11). Thus, our computational approach let us speculate that there is only a minor influence of the mutation on the EphA2–Sam/Ship2–Sam complex formation. This is not surprising considering that T1232 is not one of the Ship2–Sam residues providing the largest number of interactions with EphA2–Sam (Figure S6). 

#### 2.4.5. EphA2–Sam/A1239S Ship2–Sam Interaction

The residue A1239 is positioned at the C-terminus of the α4 helix at one edge of the Ship2–Sam ML interface (Figure S10). Out of 99 Haddock-refined structures, 96 could be collected into two clusters (Table S12). The best cluster in terms of Haddock score (i.e., first) and the most populated one (i.e., second) were analysed in detail (Figure S10). The network of intermolecular contacts characterizing the best structure of the best cluster (Figure S10a,c) appears slightly different from that of the best structure belonging to the second cluster (Figure S10b,d). For example, residues D1223 and L1225 participate in non-bonded interactions only in the best structure from the first cluster (Figure S10c). In both structures from the first and second clusters, the canonical H-bond of the Sam–Sam EH/ML complex is missing, thus pointing out that this cancer-related mutation could disturb the canonical EH/ML structural topology of binding. However, also for the EphA2–Sam/A1239S Ship2–Sam complex, Haddock scores [34] and Prodigy-predicted K_D_ values [70] do not clearly point out a large effect of the mutation on the Ship2–Sam association with EphA2–Sam (Table S11). 

Although residue A1239 has rather poor solvent accessibility and a high conservation score (Table S2), and a destabilizing effect for the A1239S substitution on the Ship2–Sam apo structure could be speculated, diverse ΔΔG predictors and molecular dynamics simulations rather point out that it could be better considered as a neutral or only poor destabilizing mutation (see also Table 3 and Section 2.2.2 and Section 2.3).

#### 2.4.6. EphA2–Sam/G1240W Ship2–Sam Interaction

Residue G1240 is positioned at the edge of the Ship2–Sam ML interface and does not belong directly to the binding interface for EphA2–Sam (Figure S11a). In fact, Ship2–Sam residue 1240 is not providing any intermolecular contact in either the wild-type (Figure S6b) or the EphA2–Sam/G1240W Ship2–Sam complexes (Figure S11b) when comparing the best Haddock-refined models from the first clusters. In this case, all 99 Haddock-refined structures could be collected into three clusters where the first cluster corresponds to the best in terms of Haddock score and also to the most populated one (Table S12). Similar to D1223G and R950W, the mutation worsens the structure convergence, leading to three conformational families instead of the two observed for the wild-type complex (Table S12). Analyses of Haddock scores [34] and Prodigy-predicted K_D_ values [70] are rather similar to those obtained for the wild-type complex (Table S11), indicating that this mutation could have a neutral effect on the affinity of EphA2–Sam for Ship2–Sam. However, G1240 is not completely solvent exposed (Table S2), and it can be speculated that the replacement of Gly 1240 with Trp could generate steric repulsions and hamper the protein stability and solubility of the isolated Ship2–Sam domain. Interestingly, this Ship2–Sam mutation, together with D1223G, induces a small local increase in flexibility according to the molecular dynamics simulations.

## 3. Materials and Methods

### 3.1. Sam Domain 3D Structure Editing 

The experimental NMR structures employed as input for computational studies on isolated Sam domains consist of the first conformers of the NMR ensembles (pdb entry codes 2E8N for EphA2–Sam and 2K4P [17] for Ship2–Sam) after the removal of flexible N- and C-terminal tails (i.e., residues 1–13 and 79–88, following the 2E8N entry sequence numbering for EphA2–Sam, and residues 22–27, according to the pdb entry 2K4P [17] for Ship2–Sam). Flexible tail removal was achieved with the “structure editing” tool of UCSF Chimera [25] (version 16.0). The resulting structures were optimized with the “Repair.pdb” macro of FoldX 4 that fixes bad torsion angles and/or Vander Waals’ clashes and optimizes side chains’ rotameric states to gain the lowest energy configurations [28]. Next, non-polar hydrogens were added to the final structures with UCSF Chimera [25]. 

### 3.2. AlphaFold2 Model Generation

AlphaFold2 [24], as implemented in the ColabFold server [77] (https://colab.research.google.com/github/sokrypton/ColabFold/blob/main/AlphaFold2.ipynb#scrollTo=kOblAo-xetgx, access date 21 June 2023), was used to predict models of wild-type EphA2–Sam (residues T908–V972 from UniProtKB [35] entry P29317 for human EphA2), EphA2–Sam I944V (residues T908–V972 with I944V mutation), and Ship2–Sam (residues G1200–K1258 from UniProtKB entry O15357 for human Ship2) as well as their cancer-related mutants (R950W, R957C, I944V–R950W, I944V–R957C for EphA2–Sam and D1223N, D1223H, D1223G, L1225M, L1228I, T1232A, E1234G, L1236M, A1239S and G1240W for Ship2–Sam). AlphaFold predictions were run without employing homologous structure templates. For each protein variant, five structures were predicted, and all of them were selected for post-prediction relaxation via gradient descent in the Amber force-field. The number of seeds was set equal to 1, and default settings were implemented for all other options. The best-ranked models for native and mutated Sam domains were next optimized by the macro “RepairPDB” of FoldX 4 [28], and non-polar hydrogens were added with UCSF Chimera [25]. 

### 3.3. Structure-Based Predictions

#### 3.3.1. Analysis of Conserved Residues

The ConSurf webserver [52] (http://consurf.tau.ac.il, access date 19 June 2023) was used to predict the evolutionary conservation of EphA2–Sam and Ship2–Sam amino acids, for which a COSMIC mutation was collected. The predictions were conducted with default parameters starting from the NMR structures, edited as described before. The ConSurf output is a score ranging from 1 to 9, where 1 and 9 indicate variable and conserved amino acids, respectively. 

#### 3.3.2. Thermodynamic Stabilities (ΔΔG Evaluation)

To evaluate the stabilizing and destabilizing effects of missense mutations, changes in the Gibbs free energies induced by mutations were evaluated by an array of predictors. ΔΔG values (expressed as ΔG Mutant − ΔG Wild-Type) were predicted with PoPMuSiC [31] (Prediction of Protein Mutant Stability Changes) (https://soft.dezyme.com/ access date, 25 June 2023), MAESTRO [32] (Multi AgEnt STability pRedictiOn) (http://biwww.che.sbg.ac.at/MAESTRO, access date 25 June 2023), and INPS-3D [33] (Impact of Non-synonymous mutations on Protein Stability) (http://inpsmd.biocomp.unibo.it, access date 25 June 2023) web servers. NMR structures (first conformers edited as indicated in Section 3.1) of EphA2–Sam, including the I944V mutation, Ship2–Sam, and the AF2 model of wild-type EphA2–Sam, were employed as input for these predictors along with lists of mutations. MAESTRO [32] ΔΔG prediction was run by setting the pH to 7. 

Additionally, ΔΔG values were predicted with FoldX 4 [28]. In this case, changes in the folding energy induced by mutations were evaluated with the “BuildModel” macro and corresponded to the “Dif_PDB” FoldX output [28,66]. For all calculations by FoldX, the default values considered for temperature, ionic strength, and pH were 298 K, 0.1 M, and pH 7, respectively [28]. 

#### 3.3.3. Thermal Stability (ΔTm Evaluation)

The HoTMuSiC web server [30] (https://soft.dezyme.com/, access date 2 June 2023) was used to predict the changes in Sam domain melting temperatures (ΔTm, expressed as Tm Mutant − Tm Wild-Type) induced by mutations. NMR structures (first conformers edited as indicated in Section 3.1) of EphA2–Sam, Ship2–Sam, and the AF2 model of EphA2–Sam were employed as input for this predictor, without employing Tm experimental values.

### 3.4. Molecular Dynamics

Ship2–Sam and EphA2–Sam NMR structures (1st conformers after deletion of flexible tails) and AlphaFold2 models for wild-type and selected mutant domains were subjected to molecular dynamics simulations for 1 µs using GROMACS 2020.3 [78] in an octahedron box solvated with TIP3P water models [79] and neutralized with Na^+^ and Cl^−^ counter-ions. PBCs (periodic boundary conditions) were employed, and in order to constrain all bond lengths, the LINCS (Linear Constraint Solver) algorithm [80] was used. In addition, an integration time step of 2 fs was applied; the particle mesh Ewald method to treat electrostatic interactions and a non-bonded cut-off for the Lennard–Jones potential were implemented [81]. Controlled temperature (T = 300 K) and pressure (p = 1 atm) were obtained through V-rescale [82] and the Berendsen [83] algorithms, respectively. Energy minimization, followed by 10 ps MD at 300 K, was achieved to relax water molecules, while protein atomic positions were harmonically restrained. Next, the temperature was gradually increased from 50 to 300 K through a six-step process, followed by a short (5 ps long) equilibration phase at 300 K under NPT (constant particle number, constant pressure, and constant temperature) standard conditions. For each Sam variant, the trajectory was run under NPT conditions without restraints for 1 µs. Trajectories were analysed with GROMACS [84], PyMOL [85], and VMD (Visual Molecular Dynamics) [86]. The simulation frames, starting from the 2500 ns simulation time, were clustered, and the structures exhibiting the lowest RMSD (root mean square deviation) relative to the other members of the most populated cluster were selected as MD representative. 

### 3.5. Modelling Mutated EphA2–Sam/Ship2–Sam Complexes

Three out of fifteen NMR conformers of the EphA2–Sam/Ship2–Sam complex (i.e., n. 3, 5, 7 from pdb entry code 2KSO [16]) were selected to model-mutated protein–protein interactions. Mutations in the EphA2–Sam/Ship2–Sam complex, except D1223H [87], were manually edited by replacing the native three-letter amino acid codes in the pdb files with those corresponding to the substituted amino acid [34]. As concerning the D1223H mutant, pdb coordinates of the 3 input conformers were modified in UCSF Chimera (version 16.0) by manually converting D1223 in histidine and selecting the most probable rotameric state [25]. The resulting pdb files were next submitted to the Refinement Interface of the Haddock web server (version 2.4) (https://wenmr.science.uu.nl/haddock2.4/refinement/, access date 15 September 2023) [34] to achieve a structure refinement in water explicit solvent in order to optimize interface geometry and energetics [73,74]. To have control over the histidine protonation state, the Haddock Refinement Interface was run through ad hoc modified “job_params.json” files, selecting “HISD” for all histidine residues. For the native EphA2–Sam/Ship2–Sam, the R950T EphA2–Sam/Ship2–Sam, the K956D EphA2–Sam/Ship2–Sam, and the EphA2–Sam/D1223H Ship2–Sam complex refinements were also conducted considering histidine residues in the charged “HIS+” state. The Haddock Refinement Interface generated 99 output structures that were subjected to a clusterization procedure. Clusterization was achieved by employing a 0.75 Å FCC (Fraction of Common Contacts) cut-off value and a minimum cluster size equal to 4. The FCC cut-off was chosen after performing several Haddock runs and ensuring the clustering of as many structures as possible. Average Haddock scores were calculated from those of the best 10 structures of the considered cluster, and associated errors were set as population standard deviation. In the case that the best cluster did not correspond to the most populated one, a second average value was obtained starting from the Haddock scores of the best structures of the most populated cluster. 

### 3.6. Analyses of the EphA2–Sam/Ship2–Sam Mutated Complexes and K_D_ Evaluation 

Prediction of the binding affinity (=dissociation constant (K_D_) values) of the EphA2–Sam/Ship2–Sam native and non-native complexes was performed with the Prodigy webserver (https://wenmr.science.uu.nl/prodigy/, access date 15 September 2023) [70] starting from the structures generated by the Haddock Refinement Interface. The K_D_ was evaluated either by employing just the best output structure of a specific Haddock cluster or as the average value over the best 10 structures of the cluster. 

The LigPlot+ (version 2.2.8) software [71,72] was employed to analyse the pattern of intermolecular contacts in each Sam–Sam complex and generate 2D diagrams of intermolecular interactions. H-bonds were found by setting 2.7 Å and 3.35 Å as cut-offs for H-acceptor and donor–acceptor distances, respectively. All non-bonded contacts were searched by setting 2.9 Å and 5.5 Å as cut-offs for minimum and maximum distances, respectively, between any atoms in any residues [71,72]. The maximum distance threshold for non-bonded contacts was chosen to have intermolecular contacts at the Sam–Sam interface, consistent with those retrieved by the Prodigy webserver [70]. Salt-bridges between positively and negatively charged residues were identified if the centroids of the side-chain-charged groups, that were evaluated by considering just heavy atoms, fell within 4.0 Å distance of each other and when at least one pair of Asp or Glu side-chain carboxyl oxygen atoms and side-chain nitrogen atoms of Arg, Lys, or His were within 4.0 Å distance [88]. 

LigPlot+ analyses were conducted employing, as input, the best structures of the best and most populated clusters. 

## 4. Conclusions

Herein, we set up a computational approach to analyse human cancer-related mutations affecting the Sam domains of the EphA2 receptor and Ship2 lipid phosphatase and gain insights on those protein variants possibly connected to the modulation of EphA2–Sam/Ship2–Sam interaction. 

Based only on ΔΔG predictors (Table 3), our strategy indicates that Ship2–Sam mutations L1228I and I1236M, regarding residues possessing low solvent exposure and good conservation scores (i.e., 6 for L1228 and 9 for L1236) (Table S2), might likely induce a destabilizing effect on the apo Sam domain. 

A few mutations, for which a clearly stabilizing or destabilizing role could not be attributed based on ΔΔG predictions (i.e., R950W EphA2–Sam; D1223H Ship2–Sam; D1223G Ship2–Sam; A1239S Ship2–Sam and G1240W Ship2–Sam), were more deeply analysed by molecular dynamics simulation and in silico interaction studies with the Haddock [34] refinement interface and the Prodigy webserver [70]. Molecular dynamics suggests that R950W EphA2–Sam, G1240W Ship2–Sam, and D1223G Ship2–Sam represent the Sam domain variants associated with the highest flexible profiles, although the differences with the native domains are not so relevant. Overall, MD simulations point out that the investigated mutations do not largely influence the conformation and dynamics of EphA2–Sam and Ship2–Sam isolated domains. 

Mutation of the Ship2–Sam residue T1232 (i.e., T1232A), which is also rather conserved and not really crucial for the association with EphA2–Sam, could be linked, according to three of the ΔΔG predictors, to a destabilizing effect. On the contrary, FoldX, which is considered a reliable ΔΔG predictor [89], points out that the T1232A mutation should not induce protein destabilization, in agreement with molecular dynamics simulations that do not show any relevant perturbation in the conformational and dynamical properties of T1232A Ship2–Sam. 

Moreover, in silico interaction studies further highlight that none of the mutations are expected to completely hamper the formation of the EphA2–Sam/Ship2–Sam complex. Based on the collected data and in agreement with results from previous experimental studies [16], mutations affecting residue D1223 in Ship2–Sam and R950 in EphA2–Sam might have a small negative and positive impact, respectively, on the binding affinity between EphA2–Sam and Ship2–Sam. 

Concerning the other Ship2–Sam mutations affecting residues in the ML interface, our results let us speculate that they might be linked to the modulation of diverse binding networks not involving EphA2–Sam.

Essentially, this study provides a fast and robust protocol, relying on a variety of in silico tools that can be employed to predict the effect of disease-related mutations on protein structure, dynamics, and interaction properties and gain some insights into the molecular mechanism at the base of their pathogenicity. This protocol can be employed to prioritize protein variants linked to the pathways of interest to be first produced by recombinant technology and experimentally investigated, thus saving time and costs connected to laboratory work. 

## Figures and Tables

**Table 1:** COSMIC [22] missense mutations of the Sam domain from the EphA2 receptor (https://cancer.sanger.ac.uk/cosmic/gene/analysis?ln=EPHA2, access date 1 June 2023). Mutations are indicated according to the sequence numbering of UniprotKB [35] entry P29317 for human EphA2. The second column (“Count”) indicates the number of samples exhibiting the corresponding mutation. The third column reports the type of tumours where the mutation has been encountered. Mutations without a reference are associated with the Sanger Institute Cancer Genome Project or taken from the ICGC/TCGA (International Cancer Genome Consortium/The Cancer Genome Atlas).

EphA2 Mutations	Count	Tumour Location and Histology
V904G	2	Prostate (Carcinoma; Adenocarcinoma)- Skin (Malignant melanoma)
R907C	1	Skin; Head neck (Malignant melanoma; Superficial spreading) [38]
R907S	1	Urinary tract; Bladder (Carcinoma)
T908M	1	Haematopoietic and lymphoid tissue (Haematopoietic neoplasm; Essential thrombocythaemia) [39]
S910F	1	Skin (Adnexal tumour; Malignant adnexal tumour; Eccrine porocarcinoma) [40]
E911K	1	Large intestine; Colon (Carcinoma; Adenocarcinoma) [41]
W912C	1	Ovary (Carcinoma; Serous carcinoma) [42]
E923K	1	Cervix (Carcinoma; Squamous cell carcinoma)
M926K	1	Biliary tract; Bile duct (Carcinoma)
A927V	2	Central nervous system; Brain (Glioma; Astrocytoma Grade IV; Glioblastoma multiforme) * [43]
A928D	1	Large intestine (Carcinoma; Adenocarcinoma) [41]
G929S	1	Large intestine (Carcinoma; Adenocarcinoma) [44]
G929C	1	Lung; Right upper lobe (Carcinoma; Adenocarcinoma)
Y930D	1	Lung (Carcinoma; Small cell carcinoma) [45]
T940I	1	(Malignant melanoma) [38]
D942Y	1	Endometrium (Carcinoma; Endometrioid carcinoma)
D942N	4	Prostate (Carcinoma; Adenocarcinoma), Skin (Malignant melanoma), Stomach (Carcinoma; Adenocarcinoma) [46,47]
D943N	1	Skin (Malignant melanoma) [47]
R950W	1	Endometrium (Carcinoma; Endometrioid carcinoma)
R957C	2	Biliary tract; Bile duct (Carcinoma; Adenocarcinoma) [48] Ovary (Carcinoma; Serous carcinoma) [42]
L965I	1	Stomach (Carcinoma; Intestinal adenocarcinoma) [49]

* The mutation was encountered in 2 samples from the same tumour location.

**Table 2:** COSMIC [22] missense mutations of the Sam domain from Ship2 (https://cancer.sanger.ac.uk/cosmic/gene/analysis?ln=INPPL1, access date 1 June 2023). Mutations are indicated according to sequence numbering of the UniprotKB [35] entry O15357 for human Ship2. Additional information from the COSMIC [22] catalogue is included in columns 2 and 3. The “Count” column indicates the number of samples exhibiting the corresponding mutation. The third column reports the type of tumour where the mutation has been found. Mutations lacking a reference are associated with the Sanger Institute Cancer Genome Project or derived from the ICGC/TCGA (International Cancer Genome Consortium/The Cancer Genome Atlas).

Ship2 Mutations	Count	Tumour Location and Histology
E1198K	1	Upper aerodigestive tract; Head neck (Carcinoma; Squamous cell carcinoma)
G1200S	1	Large intestine (Carcinoma; Adenocarcinoma) [44]
W1204C	1	Lung (Carcinoma; Adenocarcinoma)
R1206Q	1	Central nervous system; Brain (Glioma)
R1212C	1	Large intestine (Carcinoma; Adenocarcinoma)
D1223N	1	Endometrium (Carcinoma; Endometrioid carcinoma)
D1223H	1	Breast (Carcinoma; Ductal carcinoma)
D1223G	1	Endometrium (Carcinoma; Endometrioid carcinoma)
L1225M	1	Haematopoietic and lymphoid (Haematopoietic neoplasm; Acute myeloid leukaemia)
L1228I	1	Urinary tract; Bladder (Carcinoma)
T1232A	1	Lung (Carcinoma; Adenocarcinoma)
E1234G	1	Lung (Carcinoma; Squamous cell carcinoma)
L1236M	1	Large intestine (Carcinoma; Adenocarcinoma) [44]
A1239S	1	Liver (Other; Neoplasm)
G1240W	2	Lung; Middle lobe (Carcinoma; Adenocarcinoma)- Skin (Malignant melanoma)
P1244A	2	Stomach (Carcinoma; Signet ring adenocarcinoma) * [50]
K1247N	1	Endometrium (Carcinoma; Endometrioid carcinoma)
R1248H	1	Endometrium (Carcinoma; Endometrioid carcinoma)
L1251P	2	Thyroid (Carcinoma) *

* The mutation was encountered in 2 samples from the same tumour location.

**Table 3:** Prediction of changes in Sam domains stability induced by cancer-related mutations. ΔΔG values evaluated with PopMuSiC [31], Maestro [32], INPS-3D [33], and FoldX [28] are reported and indicate the differences in ΔG between mutated and unmutated reference structures. Only mutations positioned inside or close to the EH (EphA2–Sam) and ML (Ship2–Sam) interaction surfaces have been analysed. The predicted confidence (c_pred_) is also reported for Maestro data [32]; Δ(VdW) clashes refer to the difference in Van Der Waals clashes between mutated and reference non-mutated models, as estimated by FoldX [28]. ΔΔG values coloured in red represent possibly more destabilizing mutations.

**EphA2–Sam** **WT ^@^**	**PopMuSiC** **ΔΔG** **(kcal/mol)**	**Maestro** **ΔΔ** **G** **(kcal/mol)/** **c_pred_**	**INPS-3D** **ΔΔG** **(kcal/mol)**	**FoldX** **ΔΔG** **(kcal/mol)/** **Δ(VdW)**
R950W	0.00	0.05/ 0.86	0.41	0.38/ 0.01
R957C	0.55	0.6/ 0.92	0.67	1.26/ −0.06
**EphA2–Sam** 2E8N *	**PopMuSiC** **ΔΔG** **(kcal/mol)**	**Maestro** **ΔΔ** **G** **(kcal/mol)/** **c_pred_**	**INPS-3D** **ΔΔG** **(kcal/mol)**	**FoldX** **ΔΔG** **(kcal/mol)/** **Δ(VdW)**
I944V-R950W	0.09	0.11/ 0.84	0.2	−0.35/ 0.00
I944V-R957C	0.62	0.45/ 0.9	0.68	0.98/ 0.01
**Ship2–Sam** **2K4P ^Δ^**	**PopMuSiC** **ΔΔG** **(kcal/mol)**	**Maestro** **ΔΔ** **G** **(kcal/mol)/** **c_pred_**	**INPS-3D** **ΔΔG** **(kcal/mol)**	**FoldX** **ΔΔG** **(kcal/mol)/** **Δ(VdW)**
D1223N	0.19	−0.52/ 0.96	0.49	−0.34/ 0.00
D1223H	0.05	−0.88/ 0.97	0.47	−0.23/ 0.00
D1223G	0.28	−0.03/ 0.92	0.80	−0.15/ −0.01
L1225M	0.65	−0.2/ 0.96	0.02	0.47/ 0.08
L1228I	1.64	0.9/ 0.89	1.01	1.34/ 0.34
T1232A	1.45	1.08/ 0.9	1.02	−0.89/ 0.05
E1234G	0.77	0.58/ 0.92	0.58	−0.13/ 0.00
L1236M	1.23	0.87/ 0.93	1.17	0.49/ 0.42
A1239S	0.69	0.82/ 0.91	0.89	1.43/ 0.99
G1240W	2.46	0.67/ 0.86	0.61	2.90/ 1.72

^@^ The AF2 model of EphA2–Sam wild type (WT) (residue range T908–V972) was employed as input for the analysis. * The first conformer of the EphA2–Sam NMR structure after flexible regions deletion (pdb entry code 2E8N -residue range T908 to V972-) was employed as input for analysis. ^Δ^ The first conformer of the Ship2–Sam NMR structure (pdb entry code 2K4P -residue range G1200–K1258-) after N-terminal flexible region deletion was employed as input for analysis.

## Data Availability

Additional data are contained in the Supplementary Online Material or will be provided by the corresponding author upon reasonable request.

## Supplementary Materials

The following supporting information can be downloaded at: https://www.mdpi.com/article/10.3390/molecules29051024/s1. Ref. [90] is cited in supplementary file.
