# Supplementary material for: Cancer-Related Mutations in the Sam Domains of EphA2 Receptor and Ship2 Lipid Phosphatase: A Computational Study
Source: Molecules. 2024 Feb 27;29(5):1024. doi: 10.3390/molecules29051024 (PMC10935306; doi:10.3390/molecules29051024)
Supplement: Supplementary file 1 [file molecules-29-01024-s001.zip › molecules-2813317-Supplementary Figures.pdf]

Supplementary Figures

Figure S1. AF2 models of EphA2-Sam wild-type (WT) and cancer-related mutants R950W and R957C.
Figure S2. NMR structure of EphA2-Sam and AF2 models of I944V EphA2-Sam and mutants I944V-R950W, I944V-R957C.
Figure S3. NMR structure of Ship2-Sam and AF2 models of Ship2-Sam wild-type and diverse cancer-related point mutants.
Figure S4. Superimposition on the backbone atoms of EphA2-Sam NMR structure and corresponding AF2 model (I944V EphA2-Sam); overlay on the backbone atoms of AF2 models of I944V EphA2-Sam and its I944V-R950W mutant; superimposition on the backbone atoms of AF2 models of I944V EphA2-Sam and its I944V-R957C mutant.
Figure S5. The representative structures of EphA2-Sam domain variants and Ship2-Sam domain variants extracted from the MD simulations.
Figure S6. Ribbon representation of the best structure from the best Haddock cluster for the EphA2-Sam/Ship2-Sam complex. 2D diagram of intermolecular interactions generated for the best Haddock cluster of the EphA2-Sam/Ship2-Sam complex with LigPlot+.
Figure S7. Ribbon representation of the best structure from the best cluster of the R950T EphA2-Sam/Ship2-Sam complex. 2D diagram of intermolecular interactions generated by LigPlot+.
Figure S8. Ribbon representation of the K956D EphA2-Sam/Ship2-Sam complex (i.e., the best structure from the best Haddock cluster and the best structure from the most populated cluster). 2D diagrams of intermolecular interactions generated by LigPlot+.
Figure S9. Ribbon representation of the best structure from the best Haddock cluster of the EphA2-Sam/T1232A Ship2-Sam complex. 2D diagram of intermolecular interactions generated by LigPlot+.
Figure S10. Ribbon representation of the EphA2-Sam/A1239S Ship2-Sam complex (i.e., the best structure from the best Haddock cluster and the best structure from the most populated cluster). 2D diagrams of intermolecular interactions generated by LigPlot+.
Figure S11. Ribbon representation of the best structure from the best Haddock cluster of the EphA2-Sam/G1240W Ship2-Sam complex. 2D diagram of intermolecular interactions generated by LigPlot+.

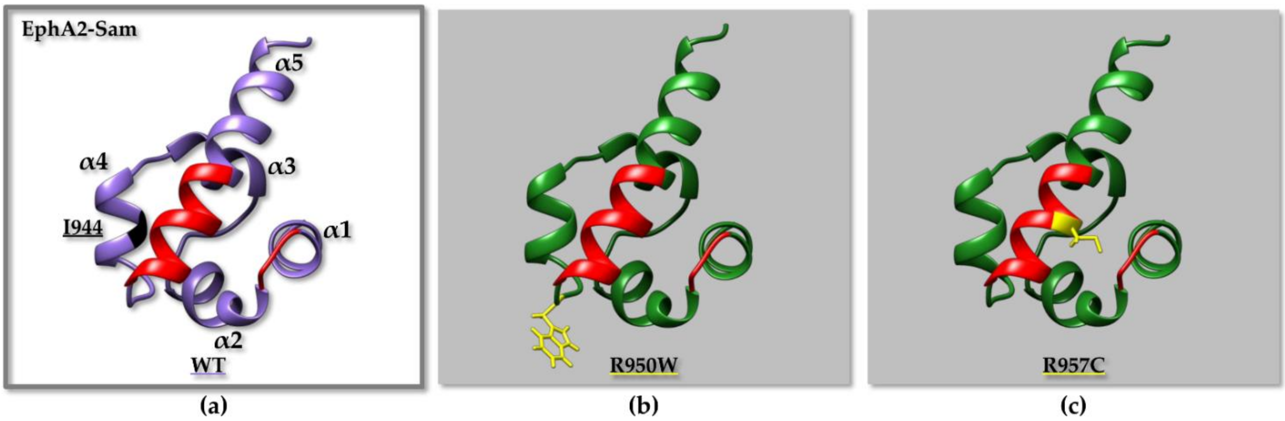

Figure S1. (**a**) AF2 [1, 2] models of EphA2-Sam wild-type (WT, residues T908-V972) and (**b, c**) cancer-related mutants (**b**) R950W and (**c**) R957C. The backbone of mutated residues is highlighted in yellow on the ribbon representations in (**b**) and (**c**) panels. The EH Interface (residues I916-M918 and P952-Y960) is coloured in red on each panel. Only the best predicted AF2 models are shown. The residue 944, that in the native sequence corresponds to an Ile, is coloured in black (**a**).

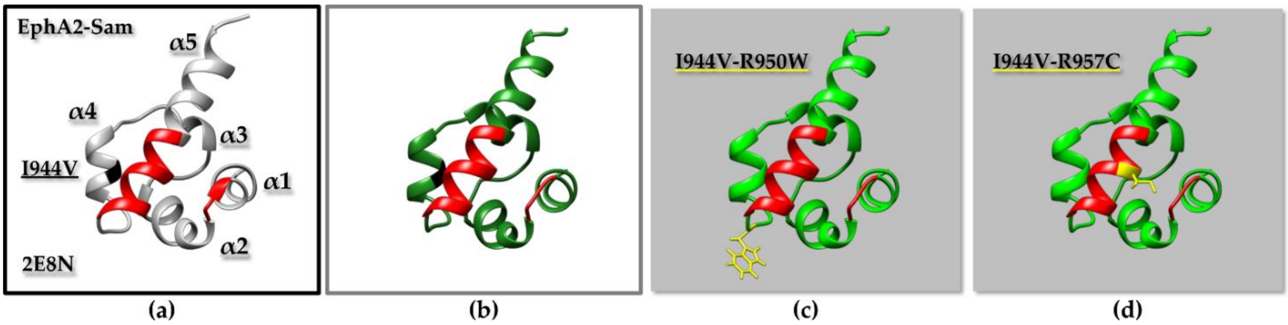

Figure S2. (**a**) NMR structure of EphA2-Sam (first conformer, pdb entry 2E8N after removal of the flexible tails, residue range T908-V972 including V944 that is highlighted in black). AF2 [1, 2] models of (**b**) I944V EphA2-Sam and cancer related mutants (**c**) I944V-R950W, (**d**) I944V-R957C. The mutated residues in cancer are highlighted in yellow on the ribbon representations in (**c**) and (**d**) panels. The EH Interface (residues I916-M918 and P952-Y960) is coloured in red in all panels. Only the best predicted AF2 models are shown.

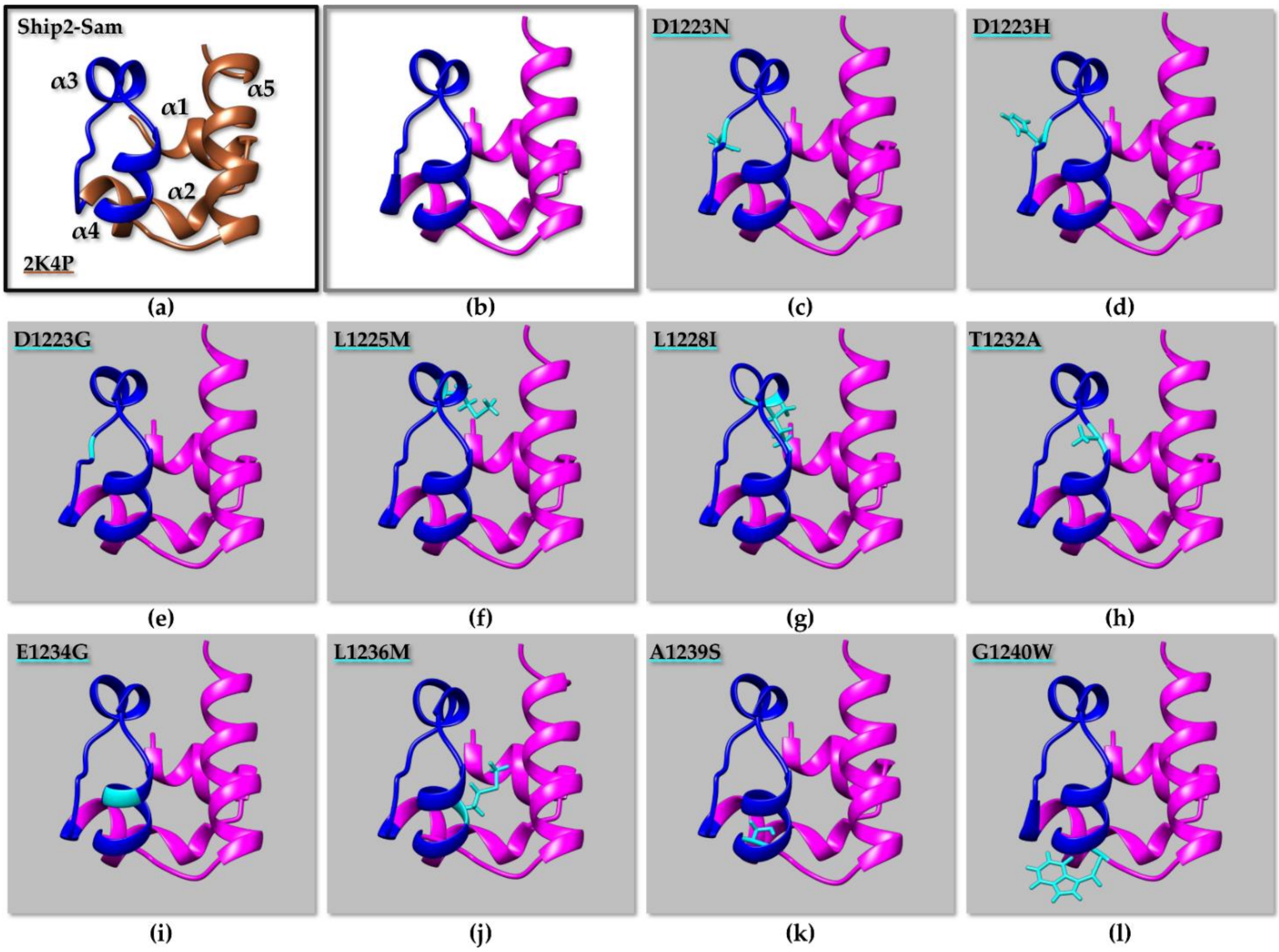

Figure S3. (**a**) NMR structure of Ship2-Sam (first conformer, pdb code 2K4P [3] after removal of the flexible N-terminal region, residue range G1200-K1258) (brown). (**b-l**) AF2 models [1, 2] of (**b**) Ship2-Sam wild-type and (**c-l**) diverse cancer-related point mutants. In each panel, the ML interface (residues H1219-E1238) is coloured in blue and the backbone and side chains of mutated residues within or close to the ML are reported in cyan. Only the best predicted AF2 models are shown.

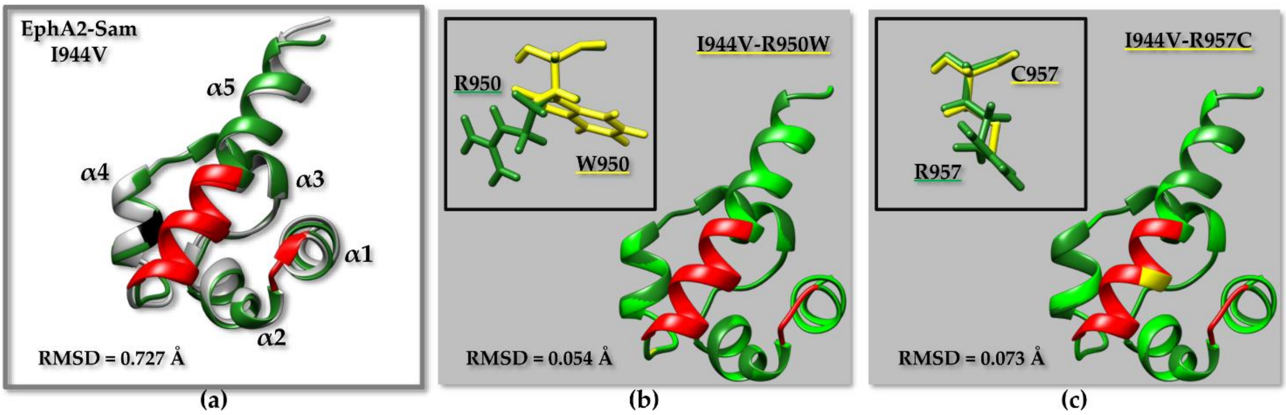

Figure S4. (**a**) Superimposition on the backbone atoms of EphA2-Sam NMR structure (first conformer, pdb entry 2E8N after removal of the flexible tails, residue range T908-V972 including V944 highlighted in black on α4) (light grey) and corresponding AF2 [1, 2] model (dark green). (**b**) Overlay on the backbone atoms of AF2 models of I944V EphA2-Sam (dark green) and its I944V-R950W mutant (light green). (**c**) Superimposition on the backbone atoms of AF2 models of I944V EphA2-Sam (dark green) and its I944V-R957C mutant (light green). The EH region of EphA2-Sam in all structures is highlighted in red. The backbone of residues, that are mutated in cancer, is coloured in yellow on the ribbon representations of the structures shown in (**b**) and (**c**). The side chains of mutated and unmutated residues are reported as well in yellow and dark green, respectively, in the upper left inserts (panels (**b**) and (**c**)). RMSD values associated with each overlay are indicated (See also Table S5).

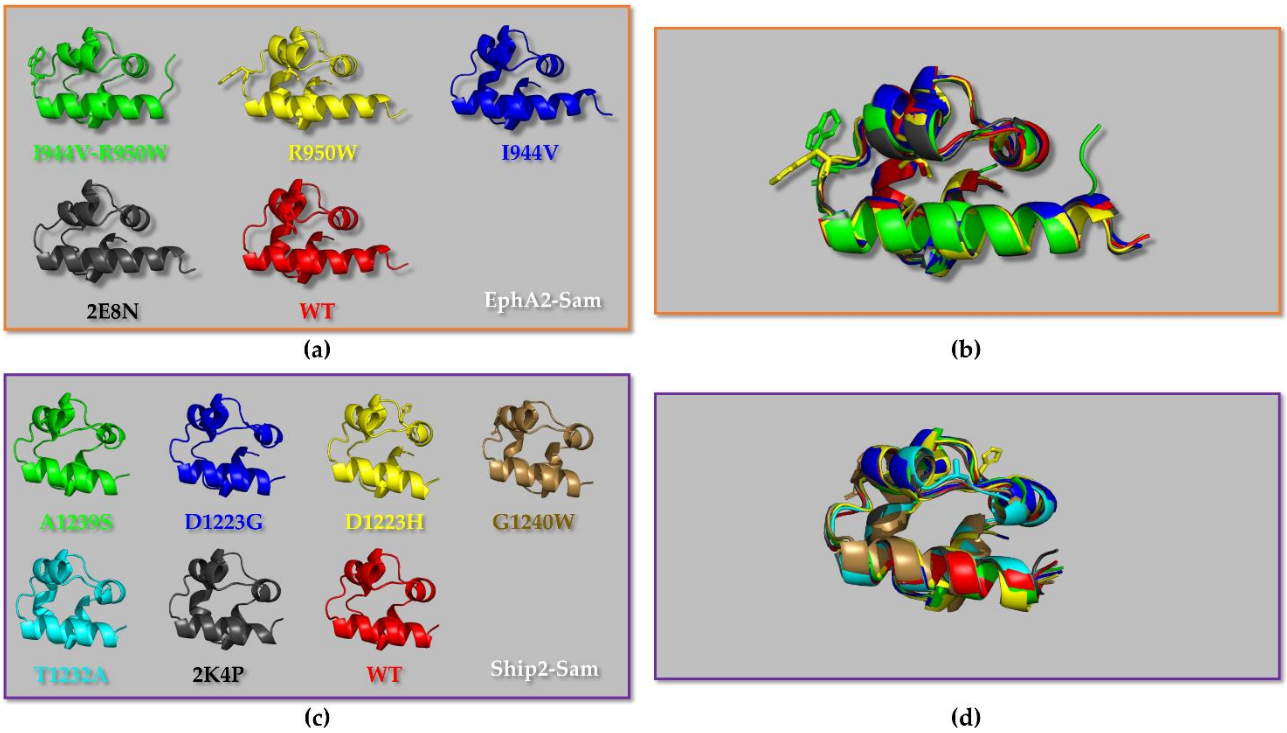

Figure S5. The representative structures of (**a**,**b**) EphA2-Sam , and (**c**,**d**) Ship2-Sam domain variants extracted from the MD simulations are shown as (**a**,**c**) separated models and (**b**,**d**) overlapped. EphA2-Sam variants are shown in cartoon representation with the following scheme color: green "I944V-R950W", yellow "R950W", blue "I944V", black "2E8N" and red "wild type". Ship2-Sam variants are shown in cartoon with the following scheme color: green "A1239S", blue "D1223G", yellow "D1223H", brown "G1240W", cyan "T1232A", black "2K4P" and red "wild type". The side chains of mutated residues are shown in stick.

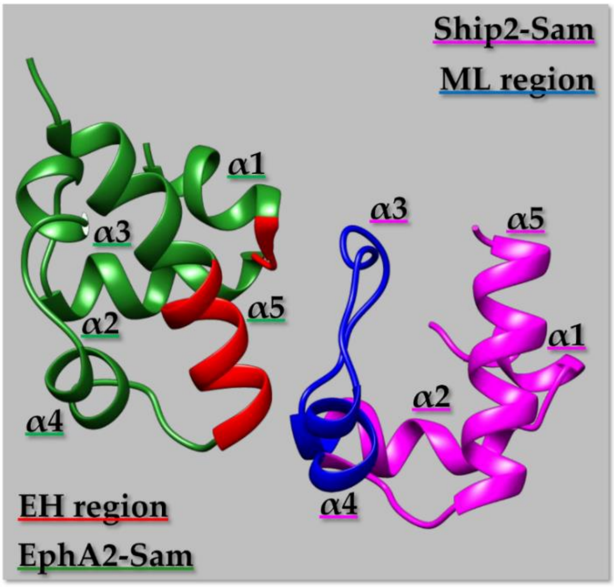

(a)

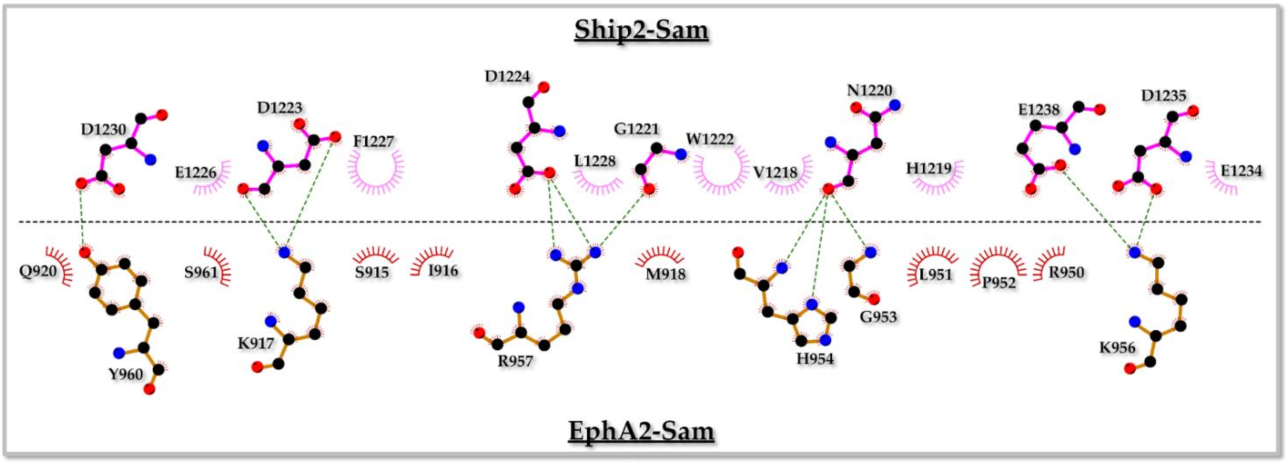

(b)

Figure S6. (**a**) Ribbon representation of the best structure from the best Haddock [4] cluster for the EphA2-Sam/Ship2-Sam complex, the EH and ML interaction interfaces in EphA2-Sam and Ship2-Sam are coloured in red and blue, respectively. (**b**) 2D diagram of intermolecular interactions generated with LigPlot+ [5, 6] for the EphA2-Sam/Ship2-Sam complex shown in (**a**). Carbon, Nitrogen, and Oxygen atoms are indicated by black, blue, and red spheres, respectively. H-bonds are highlighted with green dashed lines and were found by setting 2.7 Å and 3.35 Å as maximum cutoffs for H-acceptor and donor-acceptor distances, respectively. EphA2-Sam and Ship2-Sam residues involved in further non-bonded interactions are labeled with the one-letter amino acid code and residue number and represented by red and pink crescents with bristles, respectively.

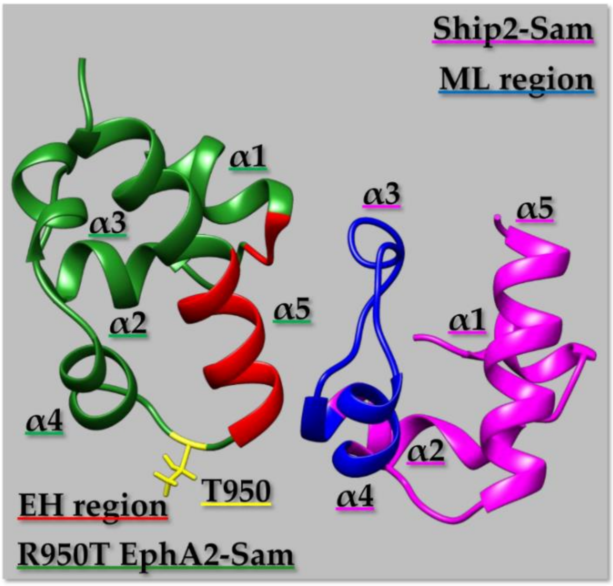

(a)

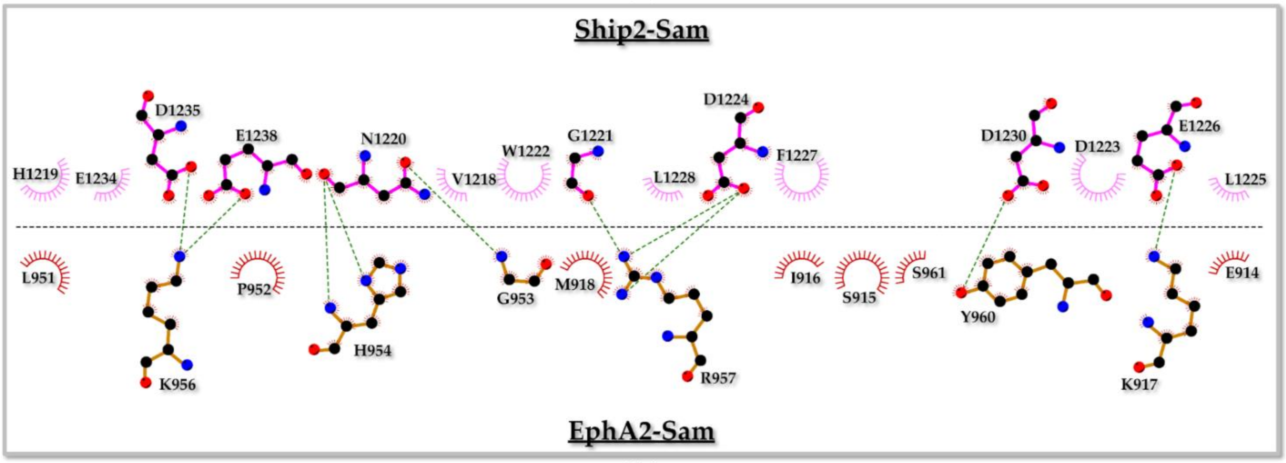

(b)

Figure S7. (**a**) Ribbon representation of the best structure from the best Haddock [4] cluster of the R950T EphA2-Sam/Ship2-Sam complex. The point mutation in Ship2-Sam is highlighted in yellow, and the EH and ML interaction interfaces in R950T EphA2-Sam, and Ship2-Sam are coloured in red and blue, respectively. (**b**) 2D diagram of intermolecular interactions generated by LigPlot+ [5, 6] for the EphA2-Sam/R950T Ship2-Sam complex (best structure from cluster 1). Carbon, Nitrogen, and Oxygen atoms are indicated by black, blue, and red spheres, respectively. H-bonds are highlighted with green dashed lines. R950T EphA2-Sam and Ship2-Sam residues involved in further non-bonded interactions are labeled and represented by red and pink crescents with bristles, respectively.

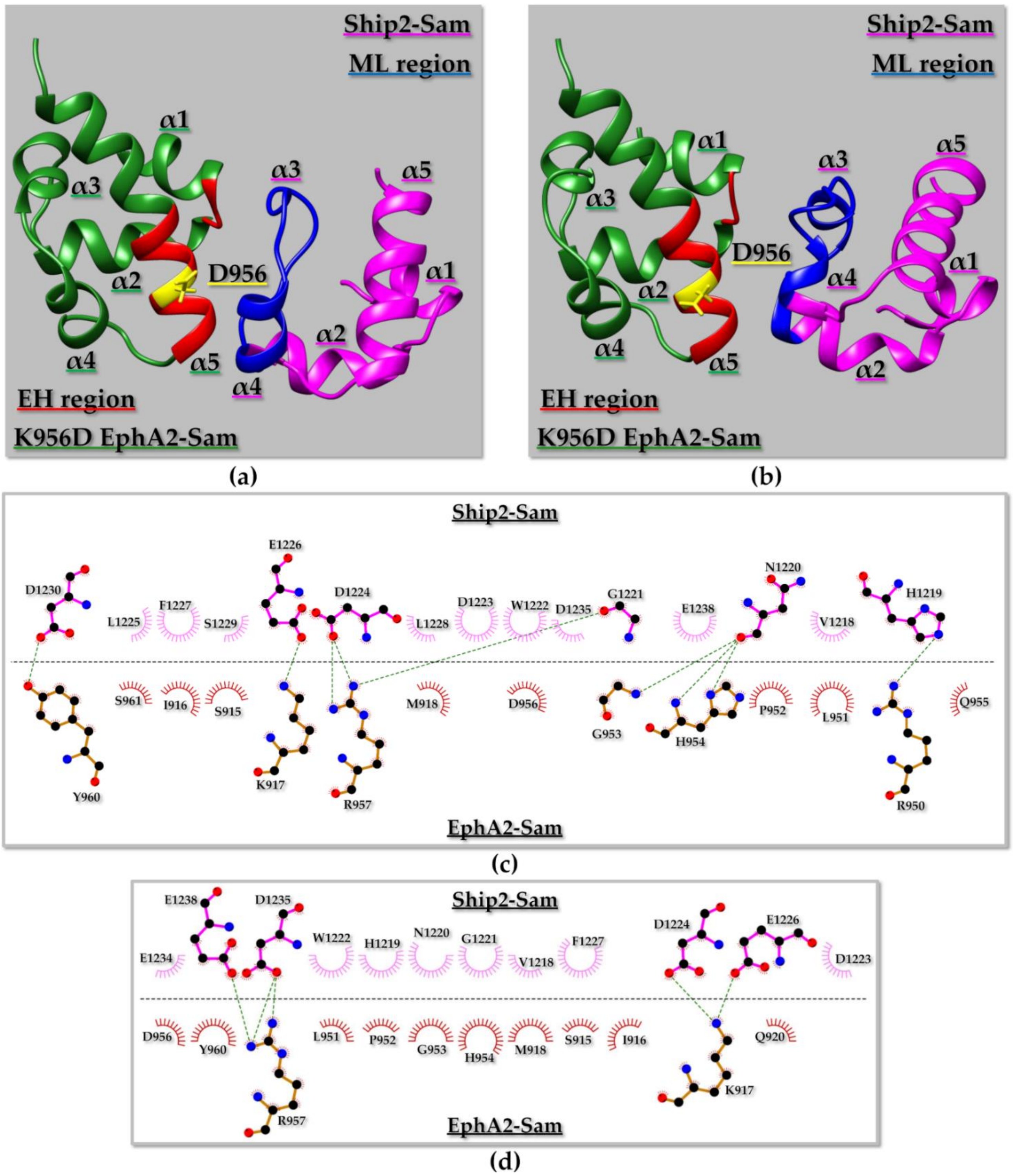

Figure S8. (**a, b**) Ribbon representation of the K956D EphA2-Sam/Ship2-Sam complex: the best structure from the best Haddock [4] cluster is shown in (**a**), whereas the best structure from the most populated cluster is shown in (**b**). The mutated residue is highlighted in yellow, and its side chain is shown as well; the EH and ML interaction interfaces in K956D EphA2-Sam, and Ship2-Sam are coloured in red and blue, respectively. (**c**) 2D diagram of intermolecular interactions generated by LigPlot+ [5, 6] analysis of the binding interface in the K956D EphA2-Sam/Ship2-Sam complex shown in panel (**a**). (**d**) 2D diagram of intermolecular contacts identified by LigPlot+ [5, 6] for the K956D EphA2-Sam/Ship2-Sam complex shown in panel (**b**). (**c, d**) Carbon, Nitrogen, and Oxygen atoms are indicated by black, blue, and red spheres, respectively. H-bonds are highlighted with green dashed lines. K956D EphA2-Sam and Ship2-Sam residues involved in non-bonded interactions are labeled and represented by red and pink crescents with bristles.

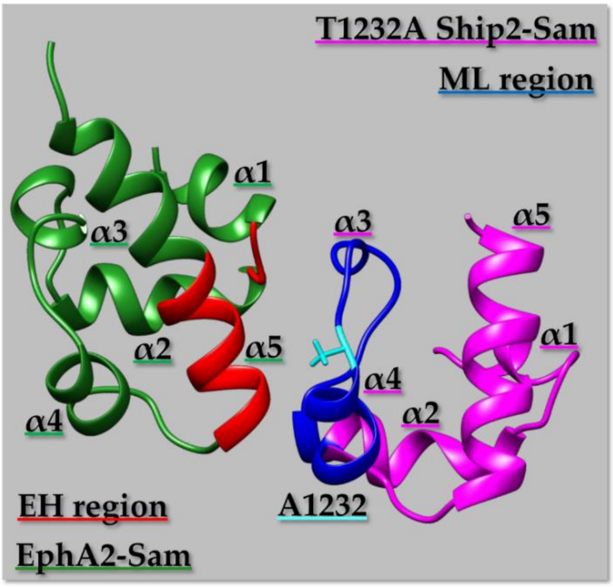

(a)

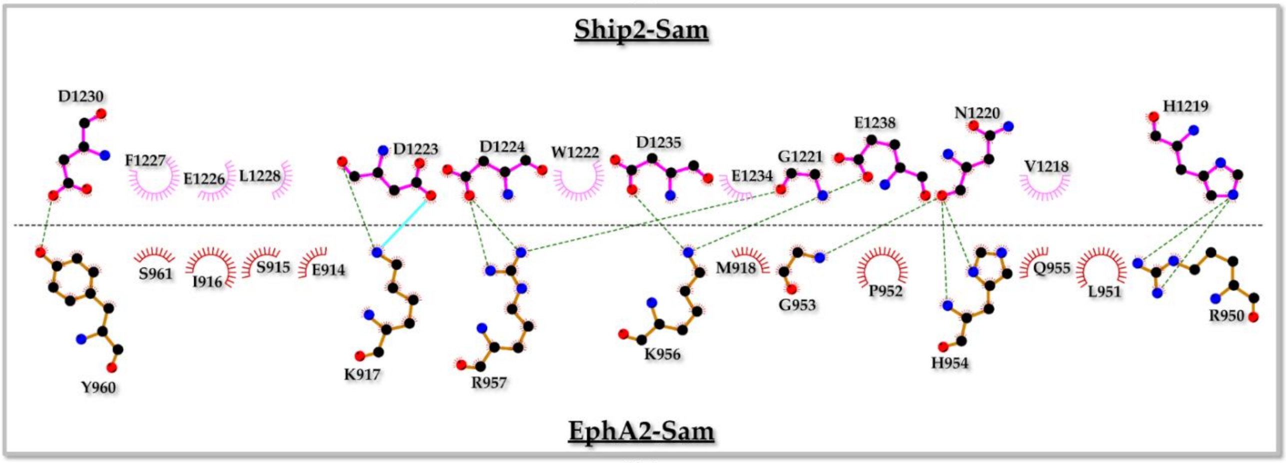

(b)

Figure S9. (**a**) Ribbon representation of the best structure from the best Haddock [4] cluster of the EphA2-Sam/T1232A Ship2-Sam complex. The point mutation in Ship2-Sam is highlighted in cyan, and the EH and ML interaction interfaces in EphA2-Sam, and T1232A Ship2-Sam are coloured in red and blue, respectively. (**b**) 2D diagram of intermolecular interactions generated by LigPlot+ [5, 6] for the EphA2-Sam/T1232A Ship2-Sam complex (best structure from best cluster). Carbon, Nitrogen, and Oxygen atoms are indicated by black, blue, and red spheres, respectively. H-bonds are highlighted with green dashed lines. A salt bridge is indicated by a cyan solid line. EphA2-Sam and T1232A Ship2-Sam residues involved in non-bonded interactions are labeled and represented by red and pink crescents with bristles, respectively.

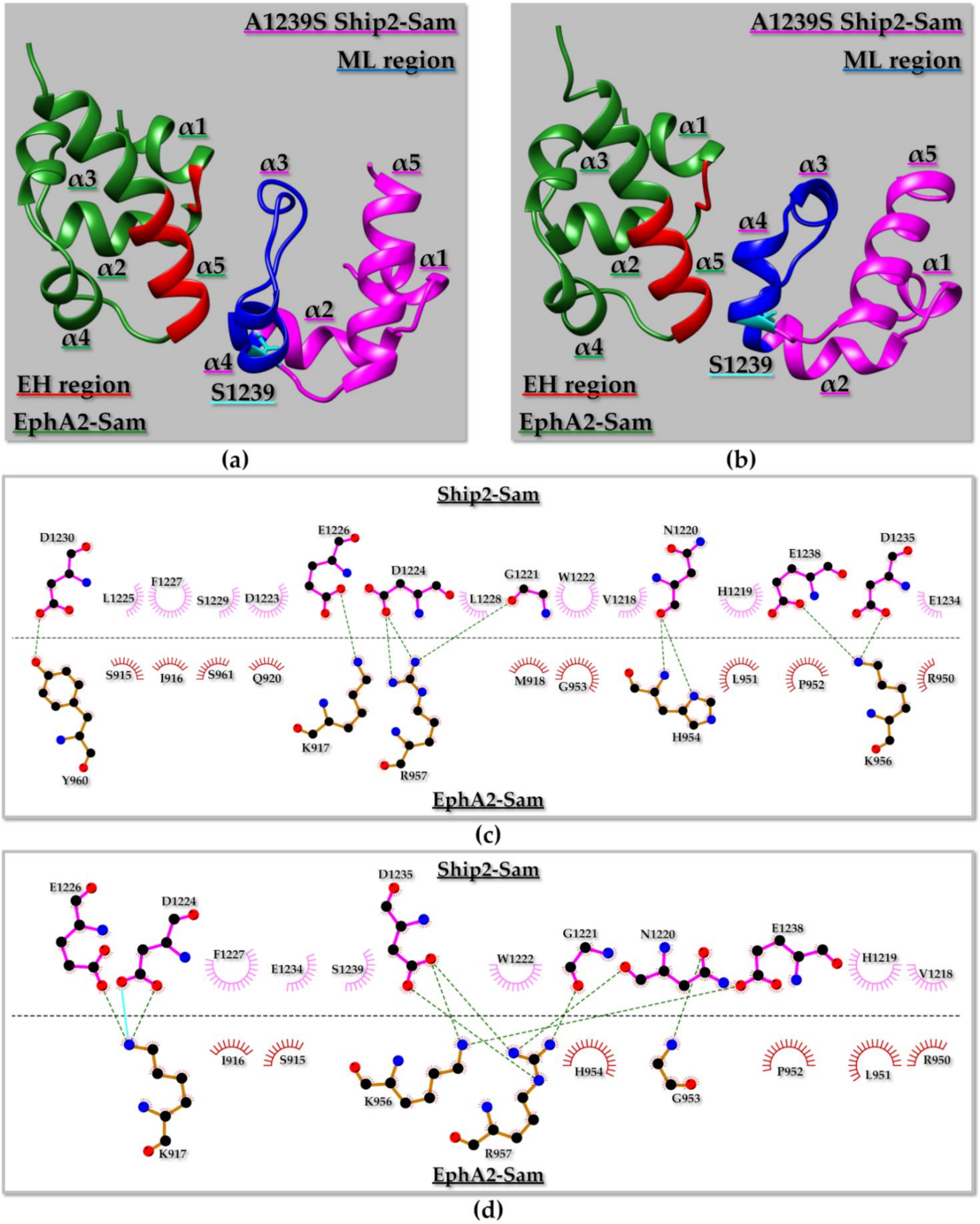

Figure S10. (**a, b**) Ribbon representation of the EphA2-Sam/A1239S Ship2-Sam complex: the best structure from the best Haddock [4] cluster is shown in (**a**), whereas the best structure from the most populated cluster is shown in (**b**). The mutated residue is highlighted in cyan, and its side chain is shown as well; the EH and ML interaction interfaces in EphA2-Sam, and A1239S Ship2-Sam are coloured in red and blue, respectively. (**c**) 2D diagram of intermolecular interactions generated by LigPlot+ [5, 6] analysis of the binding interface in the EphA2-Sam/A1239S Ship2-Sam complex shown in panel (**a**). (**d**) 2D diagram of intermolecular contacts identified by LigPlot+ [5, 6] for the EphA2-Sam/A1239S Ship2-Sam complex shown in panel (**b**). (**c, d**) Carbon, Nitrogen, and Oxygen atoms are indicated by black, blue, and red spheres, respectively. H-bonds are highlighted with green dashed lines. A salt bridge is indicated by a cyan solid line. EphA2-Sam

and A1239S Ship2-Sam residues involved in non-bonded interactions are labeled and represented by red and pink crescents with bristles.

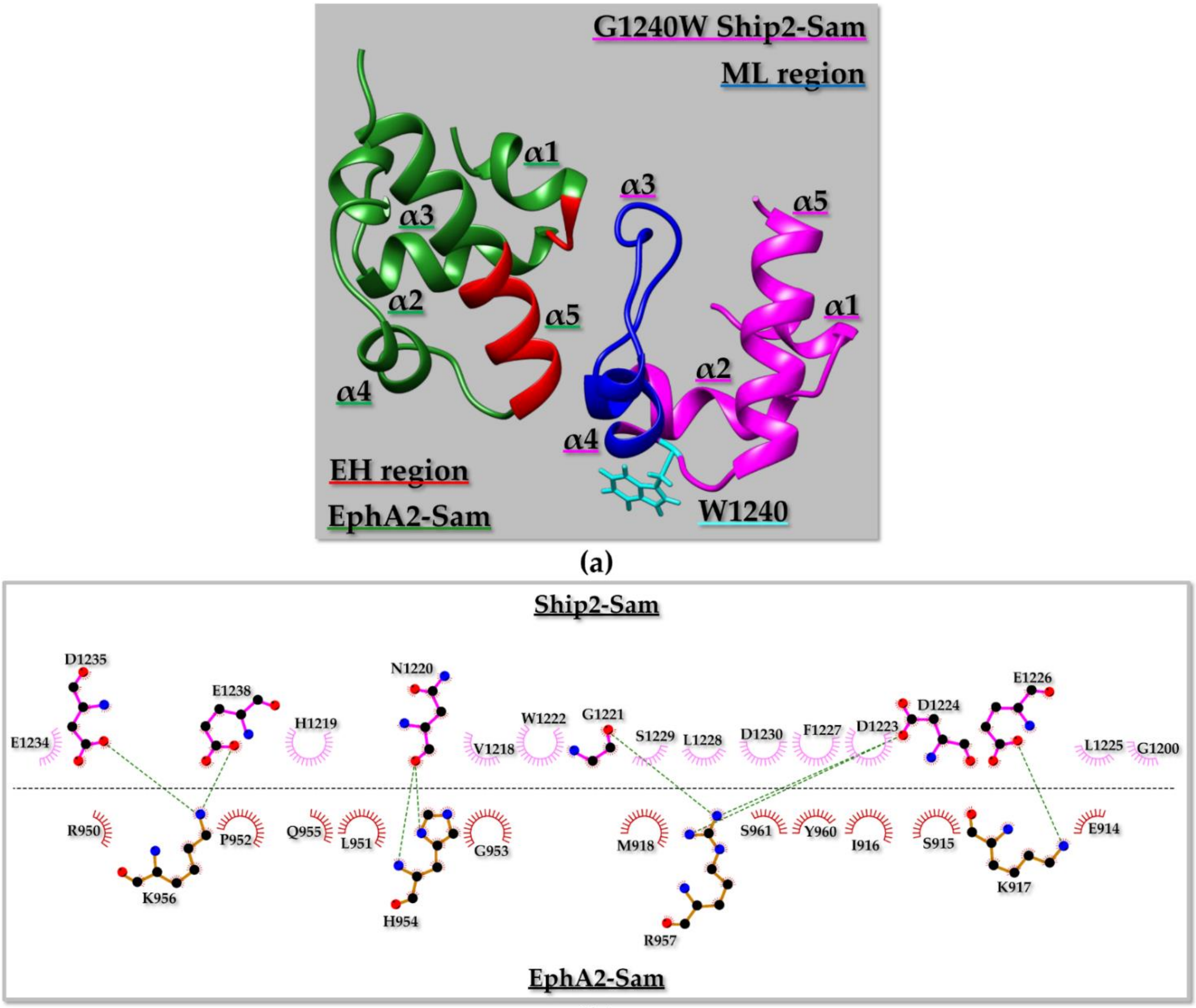

(b)

Figure S11. (**a**) Ribbon representation of the best structure from the best Haddock [4] cluster of the EphA2-Sam/G1240W Ship2-Sam complex. The point mutation in Ship2-Sam is highlighted in cyan, and the EH and ML interaction interfaces in EphA2-Sam, and G1240W Ship2-Sam are coloured in red and blue, respectively. (**b**) 2D diagram of intermolecular interactions generated by LigPlot+ [5, 6] for the EphA2-Sam/G1240W Ship2-Sam complex shown in (**a**). Carbon, Nitrogen, and Oxygen atoms are indicated by black, blue, and red spheres, respectively. H-bonds are highlighted with green dashed lines. EphA2-Sam and G1240W Ship2-Sam residues involved in non-bonded interactions are labeled and represented by red and pink crescents with bristles, respectively.

1. Jumper, J.; Evans, R.; Pritzel, A.; Green, T.; Figurnov, M.; Ronneberger, O.; Tunyasuvunakool, K.; Bates, R.; Zidek, A.; Potapenko, A.; Bridgland, A.; Meyer, C.; Kohl, S.A.A.; Ballard, A.J.; Cowie, A.; Romera-Paredes, B.; Nikolov, S.; Jain, R.; Adler, J.; Back, T.; Petersen, S.; Reiman, D.; Clancy, E.; Zielinski, M.; Steinegger, M.; Pacholska, M.; Berghammer, T.; Bodenstein, S.; Silver, D.; Vinyals, O.; Senior, A.W.; Kavukcuoglu, K.; Kohli, P.; Hassabis, D. Highly accurate protein structure prediction with AlphaFold. *Nature* **2021,** *596*(7873), 583-589.
2. Varadi, M.; Anyango, S.; Deshpande, M.; Nair, S.; Natassia, C.; Yordanova, G.; Yuan, D.; Stroe, O.; Wood, G.; Laydon, A.; Zidek, A.; Green, T.; Tunyasuvunakool, K.; Petersen, S.; Jumper, J.; Clancy, E.; Green, R.; Vora, A.; Lutfi, M.; Figurnov, M.; Cowie, A.; Hobbs, N.; Kohli, P.; Kleywegt, G.; Birney, E.; Hassabis, D.; Velankar, S.

AlphaFold Protein Structure Database: massively expanding the structural coverage of protein-sequence space with high-accuracy models. *Nucleic Acids Res.* **2022,** *50*(D1), D439-D444.

3. Leone, M.; Cellitti, J.; Pellecchia, M. NMR studies of a heterotypic Sam-Sam domain association: the interaction between the lipid phosphatase Ship2 and the EphA2 receptor. *Biochemistry* **2008,** *47*(48), 12721-8.
4. de Vries, S.J.; van Dijk, M.; Bonvin, A.M. The HADDOCK web server for data-driven biomolecular docking. *Nat. Protoc.* **2010,** *5*(5), 883-97.
5. Wallace, A.C.; Laskowski, R.A.; Thornton, J.M. LIGPLOT: a program to generate schematic diagrams of protein-ligand interactions. *Protein Eng.* **1995,** *8*(2), 127-34.
6. Laskowski, R.A.; Swindells, M.B. LigPlot+: multiple ligand-protein interaction diagrams for drug discovery. *J. Chem. Inf. Model.* **2011,** *51*(10), 2778-86.
