# Supplementary material for: Cancer-Related Mutations in the Sam Domains of EphA2 Receptor and Ship2 Lipid Phosphatase: A Computational Study
Source: Molecules. 2024 Feb 27;29(5):1024. doi: 10.3390/molecules29051024 (PMC10935306; doi:10.3390/molecules29051024)
Supplement: Supplementary file 1 [file molecules-29-01024-s001.zip › molecules-2813317-supplementary tables.pdf]

Supplementary Tables

Table S1. ConSurf conservation score and MolMol solvent accessibility estimation for EphA2-Sam residues associated with COSMIC missense mutations.
Table S2. ConSurf conservation score and MolMol solvent accessibility estimation for residues of Ship2-Sam associated with COSMIC missense mutations.
Table S3. Prediction of GRAVY, Theoretical pI, and Instability index values for wild-type human EphA2-Sam (WT), the I944V mutant and cancer-related mutants.
Table S4. Prediction of GRAVY, Theoretical pI, and Instability index values for human Ship2-Sam wild-type (WT) and mutated primary sequences.
Table S5. Comparison of 3D coordinates of wild-type and mutated EphA2-Sam and Ship2-Sam variants. RMSD values.
Table S6. ΔTm HoTMuSiC predictions.
Table S7. RMSD values and number of hydrogen bonds along MD simulations for diverse EphA2-Sam and Ship2-Sam variants.
Tables S8-S9. Validation of the computational protocol for Haddock Refinement Interface and Prodigy.
Table S10. Haddock scores and dissociation constant values by Prodigy for the EphA2-Sam/Ship2-Sam wild-type and a few mutated complexes with histidine residues in the "HIS+" protonation state.
Table S11. Haddock scores and dissociation constant values by Prodigy for the EphA2-Sam/Ship2-Sam wild-type and cancer related mutated complexes.
Table S12. Results (i.e., number of clusters and relative populations) of Haddock refinement Interface for the EphA2-Sam/Ship2-Sam native and diverse cancer related mutated complexes.

Table S1. ConSurf [1, 2] conservation score and MolMol [3] solvent accessibility estimation for EphA2-Sam residues associated with COSMIC [4] missense mutations. The input structure corresponds to the first conformer of the EphA2-Sam NMR ensemble after flexible regions deletion (pdb entry code 2E8N -residue range T908 to V972-). Mutated residues V904 and R907 are not indicated as positioned inside the flexible N-terminal tail outside the folded domain. A probe radius equal to 1.4 Å and a precision equal to 5 were used to evaluate solvent accessible surface.

EphA2-Sam	ConSurf score	MolMol solvent accessibility (%)
T908	9	43.1*
S910	1	23.1
E911	5	41.2
W912	9	9.1
E923	4	47.5
M926	3	29.8
A927	3	44.8
A928	6	20.4
G929	8	22.4
Y930	4	10.3
T940	5	32.1
D942	4	39.2
D943	8	15.7
R950	5	60.2
R957	8	36.9
L965	6	8.7

* The high solvent accessibility is also related to T908 being the first N-terminal residue in the pdb file.

Table S2. ConSurf [1, 2] conservation score and MolMol [3] solvent accessibility estimation for residues of Ship2-Sam associated with COSMIC [4] missense mutations. The input structure corresponds to the first conformer of the Ship2-Sam NMR ensemble (pdb entry code 2K4P [5]-residue range G1200-K1258-) after flexible regions deletion. Residue E1198 is not reported as included in the flexible N-terminal protein region. A probe radius equal to 1.4 Å and a precision equal to 5 were used to evaluate solvent accessible surface.

Ship2-Sam	ConSurf score	MolMol solvent accessibility (%)
G1200	8	50.2*
W1204	8	3.3
R1206	4	47.4
R1212	6	51.7
D1223	9	30.6
L1225	6	20
L1228	6	0
T1232	7	30
E1234	6	49.2
L1236	9	0
A1239	8	6.8
G1240	8	20.9
P1244	2	32.8
K1247	8	14.5
R1248	3	46
L1251	7	19.2

* The high solvent accessibility is also related to G1200 being the first N-terminal residue in the pdb file.

Table S3. Prediction of GRAVY, Theoretical pI, and Instability index values obtained with the Protparam tool of ExPASy [6, 7] (https://web.expasy.org/protparam/, access date 01/06/2023) for wild-type human EphA2-Sam (WT), the I944V mutant associated with the NMR structure (pdb code 2E8N) and cancer-related mutants. Underlined mutations represent those positioned inside or close to the EphA2-Sam EH interface.

	**EphA2-Sam[1]**	**GRAVY**	**Theoretical pI**	**Instability index**
1	WT@	-0.306	8.01	28.51
	I944V*	-0.311	8.01	29.38
2	T908M	-0.266	8.08	28.51
	I944V-T908M[#]	-0.271	8.08	29.38
3	S910F	-0.251	8.01	25.55
	I944V-S910F	-0.255	8.01	26.41
4	E911K	-0.312	9.45	27.86
	I944V-E911K	-0.317	9.45	28.72
5	W912C	-0.254	7.83	38.65
	I944V-W912C	-0.258	7.83	39.51
6	E923K	-0.312	9.45	26.71
	I944V-E923K	-0.317	9.45	27.57
7	M926K	-0.395	9.1	24.3
	I944V-M926K	-0.400	9.1	25.16
8	A927V	-0.269	8.01	26.61
	I944V-A927V	-0.274	8.01	27.48
9	A928D	-0.388	6.64	27.21
	I944V-A928D	-0.392	6.64	28.07
10	G929S	-0.312	8.01	29.82
	I944V-G929S	-0.317	8.01	30.68
11	G929C	-0.262	7.83	36.58
	I944V-G929C	-0.266	7.83	37.44
12	Y930D	-0.340	6.64	28.81
	I944V-Y930D	-0.345	6.64	29.68
13	T940I	-0.226	8.01	31.27
	I944V-T940I	-0.231	8.01	32.13
14	D942Y	-0.272	9.1	32.16
	I944V-D942Y	-0.277	9.1	33.02
15	D942N	-0.306	9.16	28.51
	I944V-D942N	-0.311	9.16	29.38
16	D943N	-0.306	9.16	35.27
	I944V-D943N	-0.311	9.16	29.38
17	R950W	-0.251	6.63	30.41
	I944V-R950W	-0.255	6.63	31.27
18	R957C	-0.198	6.63	23.5
	I944V-R957C	-0.203	6.63	24.36
19	L965I	-0.295	8.01	27.21
	I944V-L965I	-0.300	8.01	28.07

[1] Sequence numbers follow those of UniprotKB [8] entry P29317.

[@] The Wild-Type (WT) sequence refers to residues -T908 to V972 -.

[*]I944V indicates the sequence of the NMR structure (pdb entry 2E8N residues from T908 to V972 including the I944V mutation).

[#]The I944V prefix indicates double mutants including cancer related mutations in addition to the I944V mutation.

Table S4. Prediction of GRAVY, Theoretical pI, and Instability index values obtained with the Protparam tool of ExPASy [6, 7] (https://web.expasy.org/protparam/, access date 01/06/2023) using as input human Ship2-Sam wild-type (WT) and mutated primary sequences. Underlined residues are those included or close to the ML interface responsible for binding to EphA2-Sam.

Ship2-Sam[1]	GRAVY	Theoretical pI	Instability index
WT[@]	-0.451	4.29	64.35
G1200S	-0.458	4.29	64.35
W1204C	-0.393	4.29	72.97
R1206Q	-0.434	4.17	66.61
R1212C	-0.332	4.17	73.07
D1223N	-0.451	4.38	66.44
D1223H	-0.446	4.51	68.36
D1223G	-0.398	4.38	62.59
L1225M	-0.483	4.29	64.35
L1228I	-0.439	4.29	64.35
T1232A	-0.408	4.29	61.08
E1234G	-0.398	4.35	55.56
L1236M	-0.483	4.29	64.35
A1239S	-0.495	4.29	67.61
G1240W	-0.459	4.29	62.91
P1244A	-0.393	4.29	61.08
K1247N	-0.444	4.17	58.82
R1248H	-0.429	4.29	58.82
L1251P	-0.542	4.29	66.33

[1] Sequence numbers follow those of UniprotKB [8] entry O15357.

[@] The Wild-Type (WT) sequence encompasses residues G1200-K1258.

Table S5. Comparison of 3D coordinates of wild-type and mutated EphA2-Sam and Ship2-Sam domains. RMSD values were evaluated with MolMol [3] by superimposing on diverse atom sets (as reported in the various columns): the AlphaFold2 (AF2) [9, 10] models of wild-type EphA2-Sam (indicated as WT) and either EphA2-Sam NMR structure (designed as 2E8N and corresponding to the first conformer, pdb entry code 2E8N after deletion of flexible N- and C-terminal segments) or different AF2 models for point mutants (R950W and R957C) (lines 1-4); the reference AF2 [9, 10] model (I944V) and either EphA2-Sam NMR structure (2E8N) or AF2 models of I944V-R950W and I944V-R957C mutants (lines 5-8); Ship2-Sam wild type AF2 [9, 10] model (indicated as WT) with either Ship2-Sam NMR structure (first conformer, pdb entry code 2K4P [5] after deletion of N-terminal flexible region) or different mutated AF2 models (lines 9-20). "Heavy all" stands for all heavy atoms (C, N, O, S) in all residues from T908 to V972 (EphA2-Sam) and from G1200 to K1258 (Ship2-Sam) except those in mutated side chains that were excluded; "bb" stands for backbone atoms (N, Cα and C'). EH stands for End Helix interaction surface: residues I916-M918 and P952-Y960 (EphA2-Sam), and ML stands for Mid Loop interaction surface: residues H1219-E1238 (Ship2-Sam).

	**EphA2-Sam**		**RMSD (Å)**	
		**bb all**	**Heavy all**	**bb EH**
1	WT	-----------	-----------	-----------
2	2E8N	0.713	1.298	0.356
3	R950W	0.396	1.154	0.064
4	R957C	0.068	0.618	0.061
5	I944V	-----------	-----------	-----------
6	2E8N	0.727	1.250	0.348
7	I944V-R950W	0.054	0.326	0.029
8	I944V- R957C	0.073	1.085	0.061
	**Ship2-Sam**		**RMSD (Å)**	
		**bb all**	**Heavy all**	**bb ML**
9	WT	-----------	-----------	-----------
10	2K4P	1.13	1.875	0.618
11	D1223N	0.108	0.92	0.106
12	D1223H	0.227	0.494	0.189
13	D1223G	0.307	0.579	0.305
14	L1225M	0.211	0.913	0.129
15	L1228I	0.165	0.849	0.171
16	T1232A	0.147	0.874	0.138
17	E1234G	0.09	0.475	0.079
18	L1236M	0.357	0.968	0.279
19	A1239S	0.182	0.541	0.172
20	G1240W	0.128	0.319	0.119

Table S6. ΔTm HoTMuSiC [11, 12] predictions for Sam domain mutations positioned inside or close to the EH (EphA2-Sam) and ML (Ship2-Sam) interaction surfaces. HoTMuSiC values <-1 are coloured in red and are considered indicative of the most destabilizing mutations.

**EphA2-Sam WT[@]**	**HoTMuSiC ΔTm (°C)**
R950W	-2.23
R957C	-3.28
**EphA2-Sam 2E8N***	**HoTMuSiC ΔTm (°C)**
I944V-R950W	-2.53
I944V-R957C	-2.45
**Ship2-Sam 2K4P[Δ]**	**HoTMuSiC ΔTm (°C)**
D1223N	-1.51
D1223H	-0.8
D1223G	-2.01
L1225M	-1.67
L1228I	-9.16
T1232A	-6.65
E1234G	-2.47
L1236M	-6.33
A1239S	-0.58
G1240W	-5.27

[@]The AF2 model of EphA2-Sam wild-type (residue range T908-V972) was employed as input for the analysis.
*The first conformer of the EphA2-Sam NMR structure after flexible regions deletion (pdb entry code 2E8N -residue range T908 to V972-) was employed as input for the analysis.
[Δ]The first conformer of the Ship2-Sam NMR structure (pdb entry code 2K4P [5]-residue range G1200-K1258-) after flexible regions deletion was employed as input for the analysis.

Table S7. Average RMSD values for diverse EphA2-Sam and Ship2-Sam variants, evaluated along MD simulations considering Cα atoms, and number of hydrogen bonds along the MD simulations.

**RMSD (nm)**		**Number of hydrogen bonds**			
Standard Deviations	*Mean values*	*Standard Deviations*	*Mean values*		
0.08	0.22	3.6	50	**I944V-R950W**	
0.05	0.17	3.9	51	**R950W**	
0.05	0.16	3.8	51	**I944V**	**EphA2**
0.04	0.14	3.8	51	**2E8N**	
0.05	0.16	3.9	51	**WT**	
0.02	0.11	3.4	49	**A1239S**	
0.05	0.17	3.8	48	**D1223G**	
0.02	0.10	3.6	48	**D1223H**	
0.02	0.12	3.4	48	**T1232A**	**Ship2**
0.06	0.18	3.9	48	**G1240W**	
0.02	0.15	3.5	48	**2K4P**	
0.04	0.13	3.8	47	**WT**	

Table S8. Validation of the computational protocol: results (i.e., number of clusters and relative populations) of Haddock [13] refinement interface for the EphA2-Sam/Ship2-Sam native and diverse non-native complexes. The 1st cluster corresponds to the one with the best (i.e., lowest) Haddock score (evaluated as an average value over the best 4 structures) [13].

EphA2-Sam	Ship2-Sam	Cluster	Population
wt	wt	1st Haddock cluster 1	66/99
		2nd Haddock cluster 2	33/99
R950T	wt	1st Haddock cluster 1	79/99
		2nd Haddock cluster 3	7/99
		3rd Haddock cluster 2	13/99
K956D	wt	1st Haddock cluster 2	27/98
		2nd Haddock cluster 1	65/98
		3rd Haddock cluster 3	6/98

Table S9. Validation of the computational protocol: results of the Haddock refinement interface [13] and Prodigy webserver [14] for the EphA2-Sam/Ship2-Sam wild-type (wt-wt), the R950T EphA2-Sam/Ship2-Sam (R950T-wt) and the K956D EphA2-Sam/Ship2-Sam (K956D-wt) complexes. For each complex Haddock scores [13] and dissociation constants predicted by Prodigy [14] are reported for just the best cluster, if this also corresponds to the most populated one, or for both the best and the most populated clusters. The last column contains experimental (Exp.) binding data (from reference [15]).

EphA2-Sam	Ship2-Sam	Cluster	Average Haddock Score[@]	Average K_D* (μM)	Haddock Score[§]	K_D[#] (μM)	Exp. K_D (μM)
**wt**	**wt**	1st (Best & Most populated) Haddock cluster 1	-117.0 ± 2.3	1.6 ± 1.3	-121.3	1.6	5.2 ± 1.2
**R950T**	**wt**	1st (Best & Most populated) Haddock cluster 1	-114.1 ± 1.5	0.9 ± 0.2	-117.2	1.0	~4 fold enhanced binding
**K956D**	**wt**	1st (Best) Haddock cluster 2	-87.8 ± 3.1	1.8 ± 0.9	-94.5	1.1	> 50
		2nd (Most populated) Haddock cluster 1	-86.5 ± 2.8	4.5 ± 2.2	-94.0	8.0	

[@] The Haddock scores were averaged over the best 10 structures of the indicated cluster; * the K_D values were averaged over the best 10 Haddock structures of the indicated cluster; [§] the Haddock scores refer to the best structure in each cluster; [#] the K_D values refer to the best structure of each cluster.

Table S10. Haddock scores [13] and dissociation constant values by Prodigy [14] for the EphA2-Sam/Ship2-Sam wild-type and a few mutated complexes with histidine residues in the "HIS+" protonation state.

EphA2-Sam	Ship2-Sam	Cluster	Average Haddock Score[@]	Average K_D* (μM)	Haddock Score[#]	K_D[$] (μM)
wt	wt	1st (Best) Haddock cluster 2	-120.8 ± 2.6	0.7 ± 0.2	-125.9	0.6
		2nd(Most populated) Haddock cluster 1	-110.5 ± 3.8	7.0 ± 6.1	-117.6	1.0
R950T	wt	1st (Best) Haddock cluster 2	-124.2 ± 2.9	0.6 ± 0.2	-129.4	0.5
		2nd(Most populated) Haddock cluster 1	-118.3 ± 7.8	2.5 ± 2.3	-131.4	0.5
R950W	wt	1st (Best) Haddock cluster 2	-125.0 ± 2.5	0.6 ± 0.3	-129.5	0.5
		2nd (Most populated) Haddock cluster 1	-111.2 ± 3.4	5.9 ± 3.2	-118.4	2.2
wt	D1223H	1st (Best & Most populated) Haddock cluster 1	-108.8 ± 1.6	1.6 ± 0.9	-111.4	3.7

[@] The Haddock scores were averaged over the best 10 structures of the indicated cluster; * the K_D values were averaged over the best 10 Haddock structures of the indicated cluster; [#] the Haddock scores and [$] the K_D values refer to the best structure of the indicated cluster.

Table S11. Haddock scores [13] and dissociation constant values by Prodigy [14] for the EphA2-Sam/Ship2-Sam wild-type and cancer related mutated complexes.

EphA2-Sam	Ship2-Sam	Cluster	Average Haddock Score[@]	Average K_D* (μM)	Haddock Score[#]	K_D[$] (μM)
wt	wt	1st (Best & Most populated) Haddock cluster 1	-117.0 ± 2.3	1.6 ± 1.3	-121.3	1.6
R950W	wt	1st (Best) Haddock cluster 2	-116.2 ± 2.6	0.8 ± 0.3	-121.6	1.3
		2nd (Most populated) Haddock cluster 1	-115.4 ± 2.4	1.3 ± 0.9	-120.7	1.2
wt	D1223H	1st (Best & Most populated) Haddock cluster 1	-116.2 ± 2.4	1.2 ± 0.4	-119.2	1.0
wt	D1223G	1st (Best & Most populated) Haddock cluster 1	-117.3 ± 3.2	1.3 ± 0.4	-124.0	1.0
wt	T1232A	1st (Best & Most populated) Haddock cluster 1	-120.6 ± 4.1	1.5 ± 0.5	-131.6	1.6
wt	A1239S	1st (Best) Haddock cluster 2	-120.2 ± 1.9	0.9 ± 0.4	-122.9	1.2
		2nd (Most populated) Haddock cluster 1	-113.4 ± 3.0	2.9 ± 0.9	-118.9	2.0
wt	G1240W	1st (Best & Most populated) Haddock cluster 1	-120.2 ± 2.5	0.9 ± 0.4	-124.2	0.6

[@] The Haddock scores represent average values over the best 10 structures of the indicated cluster; * the K_D values were averaged over the best 10 structures of the indicated cluster; [#] the Haddock scores refer to the best structure of each cluster; [$] the K_D values were evaluated from the best structure of the indicated cluster.

Table S12. Results (i.e., number of clusters and relative populations) of Haddock [13] refinement Interface for the EphA2-Sam/Ship2-Sam native and diverse cancer related mutated complexes.

EphA2-Sam	Ship2-Sam	Cluster	Population
wt	wt	1st Haddock cluster 1	66/99
		2nd Haddock cluster 2	33/99
R950W	wt	1st Haddock cluster 2	18/99
		2nd Haddock cluster 1	70/99
		3rd Haddock cluster 3	11/99
wt	D1223H	1st Haddock cluster 1	99/99
wt	D1223G	1st Haddock cluster 1	76/99
		2nd Haddock cluster 3	6/99
		3rd Haddock cluster 2	17/99
wt	T1232A	1st Haddock cluster 1	83/97
		2nd Haddock cluster 2	14/97
wt	A1239S	1st Haddock cluster 2	33/96
		2nd Haddock cluster 1	63/96
wt	G1240W	1st Haddock cluster 1	68/99
		2nd Haddock cluster 3	7/99
		3rd Haddock cluster 2	24/99

1. Ashkenazy, H.; Abadi, S.; Martz, E.; Chay, O.; Mayrose, I.; Pupko, T.; Ben-Tal, N. ConSurf 2016: an improved methodology to estimate and visualize evolutionary conservation in macromolecules. *Nucleic Acids Res.* **2016,** *44*(W1), W344-50.
2. Ashkenazy, H.; Erez, E.; Martz, E.; Pupko, T.; Ben-Tal, N. ConSurf 2010: calculating evolutionary conservation in sequence and structure of proteins and nucleic acids. *Nucleic Acids Res* **2010,** *38*(Web Server issue), W529-33.
3. Koradi, R.; Billeter, M.; Wuthrich, K. MOLMOL: a program for display and analysis of macromolecular structures. *J. Mol. Graph.* **1996,** *14*(1), 51-5, 29-32.
4. Tate, J.G.; Bamford, S.; Jubb, H.C.; Sondka, Z.; Beare, D.M.; Bindal, N.; Boutselakis, H.; Cole, C.G.; Creatore, C.; Dawson, E.; Fish, P.; Harsha, B.; Hathaway, C.; Jupe, S.C.; Kok, C.Y.; Noble, K.; Ponting, L.; Ramshaw, C.C.; Rye, C.E.; Speedy, H.E.; Stefancsik, R.; Thompson, S.L.; Wang, S.; Ward, S.; Campbell, P.J.; Forbes, S.A. COSMIC: the Catalogue Of Somatic Mutations In Cancer. *Nucleic Acids Res.* **2019,** *47*(D1), D941-D947.
5. Leone, M.; Cellitti, J.; Pellecchia, M. NMR studies of a heterotypic Sam-Sam domain association: the interaction between the lipid phosphatase Ship2 and the EphA2 receptor. *Biochemistry* **2008,** *47*(48), 12721-8.
6. Wilkins, M.R.; Gasteiger, E.; Bairoch, A.; Sanchez, J.C.; Williams, K.L.; Appel, R.D.; Hochstrasser, D.F. Protein identification and analysis tools in the ExPASy server. *Methods Mol. Biol.* **1999,** *112*, 531-52.
7. Gasteiger, E.; Hoogland, C.; Gattiker, A.; Duvaud, S.; Wilkins, M.R.; Appel, R.D.; Bairoch, A. Protein Identification and Analysis Tools on the Expasy Server;. In *The Proteomics Protocols Handbook* J.M., W., Ed. Humana Press: 2005; pp 571-607.
8. UniProt, C. UniProt: the Universal Protein Knowledgebase in 2023. *Nucleic Acids Res.* **2023,** *51*(D1), D523-D531.

9. Jumper, J.; Evans, R.; Pritzel, A.; Green, T.; Figurnov, M.; Ronneberger, O.; Tunyasuvunakool, K.; Bates, R.; Zidek, A.; Potapenko, A.; Bridgland, A.; Meyer, C.; Kohl, S.A.A.; Ballard, A.J.; Cowie, A.; Romera-Paredes, B.; Nikolov, S.; Jain, R.; Adler, J.; Back, T.; Petersen, S.; Reiman, D.; Clancy, E.; Zielinski, M.; Steinegger, M.; Pacholska, M.; Berghammer, T.; Bodenstein, S.; Silver, D.; Vinyals, O.; Senior, A.W.; Kavukcuoglu, K.; Kohli, P.; Hassabis, D. Highly accurate protein structure prediction with AlphaFold. *Nature* **2021,** *596*(7873), 583-589.
10. Varadi, M.; Anyango, S.; Deshpande, M.; Nair, S.; Natassia, C.; Yordanova, G.; Yuan, D.; Stroe, O.; Wood, G.; Laydon, A.; Zidek, A.; Green, T.; Tunyasuvunakool, K.; Petersen, S.; Jumper, J.; Clancy, E.; Green, R.; Vora, A.; Lutfi, M.; Figurnov, M.; Cowie, A.; Hobbs, N.; Kohli, P.; Kleywegt, G.; Birney, E.; Hassabis, D.; Velankar, S. AlphaFold Protein Structure Database: massively expanding the structural coverage of protein-sequence space with high-accuracy models. *Nucleic Acids Res.* **2022,** *50*(D1), D439-D444.
11. Pucci, F.; Bourgeas, R.; Rooman, M. Predicting protein thermal stability changes upon point mutations using statistical potentials: Introducing HoTMuSiC. *Sci. Rep.* **2016,** *6*, 23257.
12. Pucci, F.; Kwasigroch, J.M.; Rooman, M. Protein Thermal Stability Engineering Using HoTMuSiC. *Methods Mol. Biol.* **2020,** *2112*, 59-73.
13. de Vries, S.J.; van Dijk, M.; Bonvin, A.M. The HADDOCK web server for data-driven biomolecular docking. *Nat. Protoc.* **2010,** *5*(5), 883-97.
14. Xue, L.C.; Rodrigues, J.P.; Kastritis, P.L.; Bonvin, A.M.; Vangone, A. PRODIGY: a web server for predicting the binding affinity of protein-protein complexes. *Bioinformatics* **2016,** *32*(23), 3676-3678.
15. Lee, H.J.; Hota, P.K.; Chugha, P.; Guo, H.; Miao, H.; Zhang, L.; Kim, S.J.; Stetzik, L.; Wang, B.C.; Buck, M. NMR structure of a heterodimeric SAM:SAM complex: characterization and manipulation of EphA2 binding reveal new cellular functions of SHIP2. *Structure* **2012,** *20*(1), 41-55.
